# Molecular Detection of *Candidatus* Scalindua pacifica and Environmental Responses of Sediment Anammox Bacterial Community in the Bohai Sea, China

**DOI:** 10.1371/journal.pone.0061330

**Published:** 2013-04-08

**Authors:** Hongyue Dang, Haixia Zhou, Zhinan Zhang, Zishan Yu, Er Hua, Xiaoshou Liu, Nianzhi Jiao

**Affiliations:** 1 State Key Laboratory of Heavy Oil Processing, Key Laboratory of Bioengineering and Biotechnology in Universities of Shandong, Centre for Bioengineering and Biotechnology, China University of Petroleum (East China), Qingdao, China; 2 State Key Laboratory of Marine Environmental Science, Xiamen University, Xiamen, China; 3 College of Life Science and Technology, Ocean University of China, Qingdao, China; J. Craig Venter Institute, United States of America

## Abstract

The Bohai Sea is a large semi-enclosed shallow water basin, which receives extensive river discharges of various terrestrial and anthropogenic materials such as sediments, nutrients and contaminants. How these terrigenous inputs may influence the diversity, community structure, biogeographical distribution, abundance and ecophysiology of the sediment anaerobic ammonium oxidation (anammox) bacteria was unknown. To answer this question, an investigation employing both 16S rRNA and *hzo* gene biomarkers was carried out. *Ca*. Scalindua bacteria were predominant in the surface sediments of the Bohai Sea, while non-*Scalindua* anammox bacteria were also detected in the Yellow River estuary and inner part of Liaodong Bay that received strong riverine and anthropogenic impacts. A novel 16S rRNA gene sequence clade was identified, putatively representing an anammox bacterial new candidate species tentatively named “*Ca*. Scalindua pacifica”. Several groups of environmental factors, usually with distinct physicochemical or biogeochemical natures, including general marine and estuarine physicochemical properties, availability of anammox substrates (inorganic N compounds), alternative reductants and oxidants, environmental variations caused by river discharges and associated contaminants such as heavy metals, were identified to likely play important roles in influencing the ecology and biogeochemical functioning of the sediment anammox bacteria. In addition to inorganic N compounds that might play a key role in shaping the anammox microbiota, organic carbon, organic nitrogen, sulfate, sulfide and metals all showed the potentials to participate in the anammox process, releasing the strict dependence of the anammox bacteria upon the direct availability of inorganic N nutrients that might be limiting in certain areas of the Bohai Sea. The importance of inorganic N nutrients and certain other environmental factors to the sediment anammox microbiota suggests that these bacteria were active for the *in situ* N transforming process and maintained a versatile life style well adapted to the varying environmental conditions of the studied coastal ocean.

## Introduction

Bacterial anaerobic ammonium oxidation (anammox, NH_4_^+^ + NO_2_^−^ → N_2_ + 2H_2_O), which involves NO_2_^−^ as electron acceptor to oxidize NH_4_^+^, is a novel N transformation pathway contributing to N_2_ production and fixed inorganic nitrogen removal in anoxic environments [1], [2]. Since being discovered a decade ago [3], anammox bacteria have been found in diverse terrestrial, freshwater, marine and engineered ecosystems [4]–[9]. Anammox represents an important component of the global N cycle, likely causing up to 50% of N_2_ production in oxygen-depleted marine waters and sediments [10], [11]. Marine sediments constitute the largest fraction of the Earth’s “surface” and a vast portion of these sediments are anoxic and thus suitable habitat for the anammox process [12], [13]. However, the sediment anammox bacterial diversity, abundance, community structure, biogeographical distribution and especially the environmental factors that may influence these fundamental ecological characteristics are still largely unsolved.

Anammox bacteria constitute a monophyletic order (*Candidatus* Brocadiales) of anaerobic chemolithoautotrophs in *Planctomycetes* phylum, with five *Candidatus* genera: Anammoxoglobus, Brocadia, Jettenia, Kuenenia and Scalindua [3], [11]. Anammox bacteria are still impossible to isolate into pure culture. Phylogenetic analysis of 16S rRNA gene sequences is the key approach for anammox bacterial taxonomy and new species identification. Molecular techniques based on 16S rRNA genes and transcripts are also the major approach for anammox bacterial ecological studies [14], which, however, are usually not very efficient due to the lack of simple and effective PCR primers [15], [16]. Because all anammox bacteria produce hydrazine by hydrazine synthase (Hzs) as a key intermediate reductant that is eventually oxidized to N_2_ by hydrazine oxidoreductase (Hzo, recently renamed as hydrazine dehydrogenase, Hdh) [11], [17], [18], the *hzs* and *hzo* genes were proposed as functional biomarkers for anammox bacterial ecological studies [15], [16], [19]–[21]. Both genes are essential to the anammox process, thus providing physiology-relevant anammox bacteria detection that may be superior to the 16S rRNA-based approaches.

Anammox bacteria are ubiquitous in O_2_-limited marine environments, such as oxygen minimum zones and sediments [11], [17], [22]. Most studies show that the marine anammox bacteria communities are composed exclusively of *Ca*. Scalindua bacteria usually with high microdiversity [22], [23]. However, several recent studies indicate that non-*Scalindua* anammox bacteria also exist in marine environments [15], [20], [21], [24]–[26]. Currently it is not clear whether the non-*Scalindua* anammox bacteria are a common component of the marine microbiota or they only occur in certain specific marine environments.

**Figure 1:**
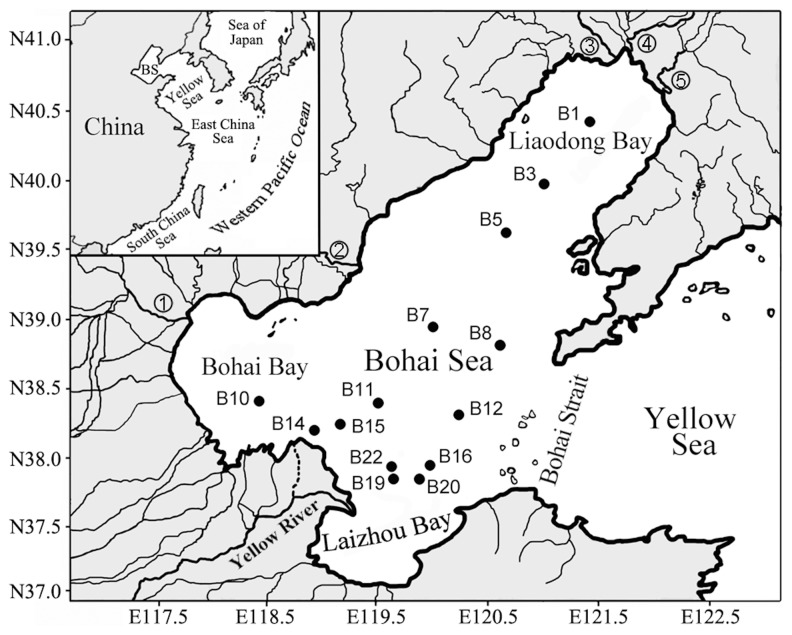
Map of the Bohai Sea and the sites of sampling stations. The insert map shows the geographical location of the Bohai Sea in the western Pacific Ocean. Abbreviation and numerical symbols: BS, Bohai Sea; 1, Haihe River; 2, Luanhe River; 3, Dalinghe River; 4, Liaohe River; and 5, Daliaohe River.

The Bohai Sea is a large semi-enclosed shallow water basin in the western Pacific Ocean, with an area of 77×10^3^ km^2^ and average water depth of only 18 m. It connects with the outer ocean (the Yellow Sea) only through the narrow Bohai Strait (Fig. 1). More than 40 rivers, including the Yellow (the fifth or the sixth longest river in the world), Liaohe, Haihe, Luanhe, Daliaohe and Dalinghe rivers, empty into the Bohai Sea with huge discharges of terrestrial materials such as sediments and nutrients. The water residence time of the Bohai Sea is about 11 years [27]. Due to the shallow depth, narrow connection with the outer ocean and slow water-exchange rate, the self-cleaning capacity of the Bohai Sea is very limited.

The Bohai Sea was once a pristine fishery ground. However, since the early 1980s, rapid industrial, agricultural and economical development and urbanization along the coastline resulted in significantly increased discharge of various pollutants into the Bohai Sea [28]. The Bohai Sea and its coast known as the Bohai Economic Rim (BER) are engaged in a variety of socioeconomic functions and services such as fisheries, aquaculture, petroleum and natural gas production, sea salt mining, hydropower generation, shipbuilding, coastal tourism and transportation. Currently, the BER (occupying only 6% of China’s total land area) contributes a quarter of the total China gross domestic product. However, most of these activities also cause pollution, especially in estuarine and coastal bay areas such as the Liaodong Bay, Bohai Bay and Laizhou Bay (Fig. 1), with major pollutants including inorganic nitrogen, organic matter, petroleum hydrocarbons and heavy metals [29]–[36]. Pollution caused by inorganic nitrogen is most serious, resulting in frequent eutrophication and harmful algal blooms [37]. According to the State Oceanic Administration of China (SOA) (http://www.soa.gov.cn/soa/hygb/hjgb/webinfo/2009/02/1271382648927217.htm), 25% area of the Bohai Sea was eutrophied in 2008 and the average seawater N/P ratio reached 67∶1, significantly higher than the established Redfield ratio (16∶1) of clean seawaters [38]. Discharges of agricultural, industrial, aquacultural and domestic wastewaters were the major causes of eutrophication. The Yellow River alone discharged ∼19×10^3^ tons of NH_4_^+^-N into the Bohai Sea in 2008 (http://www.soa.gov.cn/soa/hygbml/hq/eight/bh/webinfo/2009/08/1281687829575368.htm; SOA report). The Bohai Sea receives 47% of the total amount of the whole nation’s pollutants with an area of only 1.6% of all the China marginal seas [39]. As the most polluted and overexploited coastal ocean in China, the Bohai Sea environment and ecosystem have degraded severely. This coastal ocean may serve as a large model system to study microbial eco-functionality and pollutant biogeochemical cycling under conditions of intensive and extensive anthropogenic perturbations, to gain insightful knowledge for marine environment management and remediation.

Anammox bacteria have been detected in coastal seas of the world’s oceans and they may contribute to eutrophication reduction via removal of excess nitrogen. Several environmental factors, such as nitrite concentration, temperature, organic carbon content, organic matter mineralization rate, water depth and sedimentological status, were found to likely play major roles in shaping the sediment anammox bacteria community structure, spatial distribution, abundance and activity in marine environments [20], [40]–[42]. However, currently it is still difficult to obtain a generic view of the relationship between the sediment anammox bacterial community and environmental conditions globally. Specific habitat and environmental condition may promote the formation of unique anammox bacterial community. For example, coastal eutrophication caused by anthropogenic activities may have a strong impact on the marine anammox microbiota [20]. The pollution status of the Bohai Sea is highly complex, caused by several major types of pollutants likely with distinct respective sources and spatial distributions. How these pollutants, along multiple spatial gradients, may influence the diversity, community structure, spatial distribution, abundance and ecophysiology of the sediment anammox bacteria is currently unknown.

In this study, an ocean-wide ecological investigation of the sediment anammox bacterial communities and the *Ca*. Scalindua assemblages in particular was carried out in the Bohai Sea. The influence of key environmental factors including major environmental pollutants on the sediment anammox microbiota was also analyzed. A putative new candidate species of the anammox bacteria and versatile environmental adaptivity of the *in situ* anammox microbiota were identified.

## Materials and Methods

### Ethics statement

No specific permit was required for the described field study.

### Sample collection and environmental factor measurements

Sediment samples were collected in a cruise of R/V ‘Dong Fang Hong 2′ in August 2008 from 14 sampling stations, covering Bohai Sea typical environments including the Yellow River estuary and Liaodong Bay (Fig. 1). Sediments were collected with a stainless steel 0.1 m^2^ Gray O’Hara box corer and only undisturbed core samples with clear overlying water were used [43]. Replicate surface sediment subcore samples down to 5-cm depth for microbiological and environmental analyses were taken with sterile 60 ml syringes (luer end removed), homogenized and stored in airtight sterile plastic bags at –20°C during the cruise and –80°C after returning to the laboratory.

At each station, seawater physicochemical and biological parameters, including water depth, temperature, salinity, EC25 (electrical conductivity calibrated at water temperature of 25°C), density, turbidity and chlorophyll *a* (Chl-*a*), were measured *in situ* at various water depths with a Compact-CTD equipped with a TCDKU sensor (Alec Electronics, Japan) (Table S1). Other environmental parameters were measured in the laboratory. Sediment chloroplast pigment contents were determined as Chl-*a* and phaeophorbide *a* (Pha-*a*) with a RF-5301PC spectrofluorophotometer (Shimadzu, Japan) following a previous protocol [44]. Sediment organic carbon (OrgC) and organic nitrogen (OrgN) contents were measured with a PE 2400 Series II CHNS/O elemental analyzer (Perkin Elmer, Norwalk, CT, USA) [45]. Sediment contents of water, total organic matter (OM), total phosphorus (TP), inorganic phosphorus (IP) and organic phosphorus (OrgP) were measured according to Danovaro (2009) [43]. Sediment pore-water was extracted by centrifugation at 4°C [46]. Pore-water pH and *Eh* were measured with a S20 SevenEasy™ pH meter (Mettler-Toledo, Columbus, OH, USA), salinity measured with a S/Mill-E hand-held seawater salinity refractometer (Atago, Japan) and dissolved oxygen content (DO) and conductivity measured with a SensION 156 portable multi-parameter meter (Hach, Loveland, CO, USA). Concentrations of sediment pore-water nitrate (NO_3_^−^), nitrite (NO_2_^−^), ammonium (NH_4_^+^), phosphate (PO_4_^3−^) and silicate (SiO_3_^2−^) were measured with a nutrient QUAATRO AutoAnalyzer (Bran+Luebbe, Germany) [45]. Sediment pore-water sulfate (SO_4_^2−^) concentrations were measured using a Dionex ICS-90 ion chromatography system (Thermo Scientific, Waltham, MA, USA) [47]. Sediment sulfide and total petroleum hydrocarbon contents were measured, respectively, using a UV-1800 UV-VIS spectrophotometer (Shimadzu, Japan) [48], [49]. Sediment Cd, Co, Cu, Fe, Mn, Ni, Pd and Zn concentrations were measured with an AA240FS atomic absorption spectrometer (Varian, Palo Alto, CA, USA), Cr measured with a 725-ES inductively coupled plasma-optical emission spectrometer (Varian), and As and Hg measured with an AFS-820 atomic fluorescence spectrometer (Beijing Titan Instruments, China) [49], [50]. Blank and the certified reference geostandard sample GBW 07314 from SOA were used for analytical quality control. The detection limit for Hg was 1.0 ng/g, for Cd was 10.0 ng/g, and for the remaining elements were 0.2–1.0 µg/g, respectively. A Cilas 940L laser granulometer (Company Industrielle des Lasers, France) was used for sediment granularity analyses (Table S1) [45].

### DNA extraction and 16S rRNA and *hzo* gene clone library analyses

Total environmental genomic DNA was extracted from 0.3 g of sediments using a Fastprep DNA Extraction Kit for Soil and a FastPrep-24 Cell Disrupter (MP Biomedicals, Solon, OH, USA) as described previously [20], [45]. The Fastprep method is one of the most successful and efficient sediment DNA extraction methods with reasonably good DNA quality and quantity and the modified Fast+ method was followed in our current experiment except that the poly-adenylic acid was not added [51]. The PCR primers Brod541F and Brod1260R targeting specifically the 16S rRNA gene sequences of the *Ca*. Scalindua group [5] and the PCR primers *hzo*F1 and *hzo*R1 targeting specifically the cluster 1 *hzo* gene sequences of all known anammox bacterial groups [19], [21], [52] were used respectively for 16S rRNA and *hzo* gene amplifications following previous protocols [20]. PCR products from 10 or more reactions (each of 12.5 µl volume) were pooled to minimize PCR bias, gel purified, ligated into pMD19-T Simple vectors (Takara, Japan), and transformed into *Escherichia coli* TOP10 competent cells [20], [53]. Plasmid insert-positive recombinants were selected using X-Gal-IPTG LB indicator plates amended with 100 µg/ml ampicillin. A miniprep method was used to isolate plasmids [54] and the cloning vector primers RV-M and M13-D were used to reamplify the cloned DNA fragments [53]. The resulting PCR products were screened for correct size and purity by electrophoresis using 1% agarose gels.

Amplicons of correct size obtained from 16S rRNA and *hzo* gene amplifications were digested separately with *Msp*I, *Hha*I and *Taq*I endonucleases (Fermentas, Glen Burnie, MD, USA). Restriction fragments were resolved by electrophoresis on 3% agarose gels in 0.5×Tris-borate-EDTA buffer. Band patterns digitally photographed with an AlphaImager HP system (Alpha Innotech, Santa Clara, CA, USA) were compared for restriction fragment length polymorphism (RFLP) analysis to identify identical clones [20].

Plasmid inserts were sequenced with cloning vector primers M13-D and RV-M with an ABI 3770 automatic sequencer (Applied Biosystems, Foster City, CA, USA). Possible chimerical DNA sequences were checked with programs CHIMERA_CHECK, Bellerophon and Pintail [55]–[57]. Top-hit GenBank 16S rRNA gene and Hzo protein sequences were retrieved using the BLAST program [58]. The 16S rRNA gene sequences were grouped into operational taxonomic units (OTUs) with 0.5% distance cutoff and the Hzo protein sequences were grouped with 1% distance cutoff using the DOTUR program [59]. Small distance cutoffs were used because microdiversity among environmental anammox bacteria has been reported [20], [23]. Phylogenetic analyses followed previous procedures [20] by using CLUSTAL X (version 2.0) for sequence alignments and PHYLIP (version 3.69) for phylogenetic tree constructions [60], [61].

### Quantification of the sediment 16S rRNA and *hzo* genes

Plasmids carrying bacterial 16S rRNA, *Ca.* Scalindua 16S rRNA or anammox bacterial *hzo* gene fragments constructed previously were extracted from *E. coli* hosts using a plasmid mini kit (Qiagen, Valencia, CA, USA) and linearized with an endonuclease specific in the vector region [20], [53], [62], [63]. Linearized plasmid DNA and sediment genomic DNA concentrations were measured using PicoGreen (Molecular Probes, Eugene, OR, USA) and a Modulus single-tube multimode-reader fluorometer (Turner Biosystems, Sunnyvale, CA, USA). All real-time fluorescence quantitative PCR (qPCR) assays targeting the 16S rRNA or *hzo* genes were carried out in triplicate with an ABI Prism 7500 sequence detection system (Applied Biosystems, Foster City, CA, USA) using established SYBR green qPCR methods [20]. Agarose gel electrophoresis and melting-curve analysis were employed to confirm qPCR specificities. Standard curves were generated with serially diluted standard plasmids containing target 16S rRNA or *hzo* gene fragments. In all experiments, negative controls containing no template DNA were subjected to the same qPCR procedure to detect and exclude any possible contamination or carryover.

Sediment bacterial 16S rRNA genes were quantified using qPCR primers 341F and 518R [20], *Ca*. Scalindua 16S rRNA genes were quantified using primers Brod541F and Brod1260R [5], [20], [41] and anammox bacterial *hzo* genes were quantified using primers *hzo*F1 and *hzo*R1 [20]. The qPCR reaction conditions, thermocycling parameters and data collection procedures followed a previous publication [20].

### Statistical analyses

Coverage of each clone library was calculated as *C* =  [1– (n_1_/N)]×100, where n_1_ is the number of unique OTUs and N the total number of clones in a library [64]. Gene diversity indices (Shannon-Weiner *H*, Simpson *D* and evenness *J*) were calculated using OTUs in each library. Rarefaction analysis and two richness estimators, abundance-based coverage estimator (*S*_ACE_) and bias-corrected Chao1 (*S*_Chao1_), were calculated using program DOTUR [20], [59].

Community classification of the sediment anammox bacteria assemblages was performed with Jackknife environment clustering and principal coordinates analysis (PCoA) using the Fast UniFrac program [65]. Correlations of the anammox bacteria assemblages with environment factors were explored using canonical correspondence analysis (CCA) with statistic software CANOCO (version 4.5, Microcomputer Power, Ithaca, NY, USA) following previously procedures [20]. Pearson correlation analyses of sediment bacteria 16S rRNA, *Ca*. Scalindua 16S rRNA and anammox bacteria *hzo* gene abundances with environmental factors were performed using statistic software MINITAB (release 13.32; Minitab Inc., State College, PA, USA).

### Nucleotide sequence accession numbers

The determined partial 16S rRNA gene sequences have been deposited in GenBank under accession numbers JX537267 to JX537328 and the partial *hzo* gene sequences under accession numbers JX537329 to JX537479.

## Results

### Diversity of the *Ca*. Scalindua and the total anammox bacteria

To test the within-site variability of the *Ca*. Scalindua assemblages, two *Ca*. Scalindua 16S rRNA gene clone libraries (B22-I and B22-II) were constructed each from a separate sediment subcore sample of station B22. These two libraries appeared to be slightly different based on rarefaction analysis (Fig. S1a). However, community classification using environmental clustering (Fig. S2a) and PCoA (Fig. S3a) showed that they were similar, indicating the reproducibility of our experimental procedures and the negligible within-site variation of the *Ca*. Scalindua assemblages. Thus, these two clone libraries were combined into a single B22 library. The two anammox bacterial *hzo* gene clone libraries (B22-I and B22-II) constructed each from a separate sediment subcore sample of station B22 also showed the experimental reproducibility and negligible within-site variation (Fig. S1b, S2b and S3b), thus these two libraries were combined into a single B22 library. The observed negligible within-site variation of the *Ca*. Scalindua and total anammox bacterial assemblages makes the subsequent among-site ecological statistics valid and meaningful.

Of the 14 *Ca*. Scalindua 16S rRNA gene clone libraries constructed each for a distinct sampling station, a total of 1632 insert-positive clones were identified, resulting in 62 unique RFLP sequence types and 45 OTUs. The values of library coverage (*C*) ranged from 92.7% to 99.1% (Table 1), which together with rarefaction analysis (Fig. S1a) suggested that the *Ca*. Scalindua bacteria were sufficiently represented in these clone libraries. Station B3 had the highest diversity and station B20 had the lowest diversity based on the *H* and *J* indices, while the *S*_ACE_ and *S*_Chao1_ estimators indicated that station B22 had the highest richness and station B16 had the lowest richness of the *Ca*. Scalindua bacteria in the Bohai Sea sediments (Table 1). The combination of the B22-I and B22-II 16S rRNA gene clone libraries may increase the OTU richness of station B22 likely due to the increased detection of rare sequences. The *Ca*. Scalindua bacteria diversities mainly correlated negatively with seawater salinity, conductivity and sediment pore-water conductivity and positively with sediment pore-water nitrite and nitrate concentrations and NO_x_^−^/NH_4_^+^ (Table S2).

**Table 1:** Biodiversity and predicted richness of the sediment *Scalindua* 16S rRNA and anammox *hzo* gene sequences recovered from the sampling stations of the Bohai Sea.

Station	No. of clones	No. of gene sequences^a^	No. of OTUs*^b^*	*C* (%)	*H*	1/*D*	*J*	*S*_ACE_	*S*_Chao1_
B1	111/92*^c^*	12/21	11/19	95.5/93.5	1.87/3.52	2.74/8.85	0.54/0.83	16.5/24.6	14.3/21.5
B3	107/96	7/33	6/24	98.1/90.6	1.46/3.79	2.37/9.96	0.57/0.83	8.6/34.1	6.5/29.1
B5	131/97	12/24	10/16	96.2/94.8	1.63/3.26	2.45/7.71	0.49/0.81	17.7/21.2	13.3/19.3
B7	118/109	13/13	11/12	96.6/99.1	2.26/2.78	3.64/4.64	0.65/0.78	18.8/12.5	13.0/12.0
B8	104/96	8/23	8/16	96.2/90.6	1.63/2.83	2.46/4.59	0.54/0.71	19.0/30.6	11.0/52.0
B10	124/96	14/18	13/15	92.7/95.8	1.73/2.65	2.52/4.12	0.47/0.68	37.8/17.8	25.0/15.9
B11	115/102	13/26	11/18	95.7/92.2	1.95/3.36	2.87/8.53	0.56/0.81	18.1/32.3	16.0/27.3
B12	109/92	7/16	7/14	99.1/95.7	1.82/2.81	2.90/5.14	0.65/0.74	7.6/16.5	7.0/15.5
B14	119/86	9/14	9/14	96.6/91.9	1.56/2.18	2.39/2.53	0.49/0.57	13.7/26.4	10.5/21.0
B15	106/88	13/16	12/11	95.3/96.6	1.97/2.34	2.71/3.34	0.55/0.68	17.7/13.5	14.0/12.0
B16	90/88	7/25	6/17	98.9/90.9	2.09/3.07	3.79/5.78	0.81/0.75	6.8/34.6	6.0/24.0
B19	93/92	8/18	8/12	98.9/94.6	2.25/2.49	3.72/3.94	0.75/0.69	8.6/19.7	8.0/15.3
B20	98/121	15/19	13/18	94.9/93.4	2.95/2.85	6.54/4.90	0.80/0.68	21.4/26.7	18.0/23.6
B22	207/235	17/21	15/15	96.1/99.1	2.43/2.44	4.03/3.94	0.62/0.63	42.9/16.6	43.0/15.1

a Unique anammox 16S rRNA and *hzo* gene sequences were determined *via* RFLP analyses. *^b^* OTUs of the *Scalindua* 16S rRNA gene sequences were determined at 0.5% distance cutoff and OTUs of the anammox *hzo* gene sequences were determined at 1% distance cutoff using the DOTUR program. The coverage (*C*), Shannon-Weiner (*H*), Simpson (*D*) and evenness (*J*) indices, and S_ACE_ and S_Chao1_ richness estimators were calculated using the data of OTUs. *^c^* Clone, index and estimator data are presented for the “*Scalindua* 16S rRNA gene/anammox *hzo* gene” sequences.

Of the 14 *hzo* gene clone libraries constructed, a total of 1490 insert-positive clones were identified, resulting in 151 unique RFLP sequence types and 70 OTUs. The values of library coverage (*C*) ranged from 90.6% to 99.2% (Table 1), which together with rarefaction analysis (Fig. S1b) suggested that the anammox bacteria were sufficiently represented in these clone libraries. Station B3 had the highest diversity and station B14 had the lowest diversity based on the majority of the *H*, 1/*D* and *J* indices, while stations B16 (based on *S*_ACE_) and B8 (based on *S*_Chao1_) had the highest richness and station B7 (based on *S*_ACE_ and *S*_Chao1_) had the lowest richness of the anammox bacteria in the Bohai Sea sediments (Table 1). The *hzo*-harboring anammox bacteria diversities mainly correlated negatively with surface water turbidity, bottom water temperature, sediment OrgC, IP, TP, As and Mn contents and positively with sediment Cd and Pb contents (Table S2).

### Phylogeny of the *Ca*. Scalindua 16S rRNA gene sequences

The obtained 62 distinct *Ca*. Scalindua 16S rRNA gene sequences were 94.1–99.9% identical with one another, and 98.8–100.0% identical to the top-hit GenBank sequences, most of which (90.3%) were originally retrieved from marine sediments or anoxic seawater [5], [20], [23], [66]–[70], indicating a marine source environment of most of the Bohai Sea sequences. More than two third of the top-hit GenBank sequences (71.0%) were originally retrieved from the sediments of Jiaozhou Bay, a highly eutrophied coastal bay in China [20], likely indicating similar *Ca*. Scalindua assemblages of these two highly human-activity-impacted marine environments.

**Figure 2:**
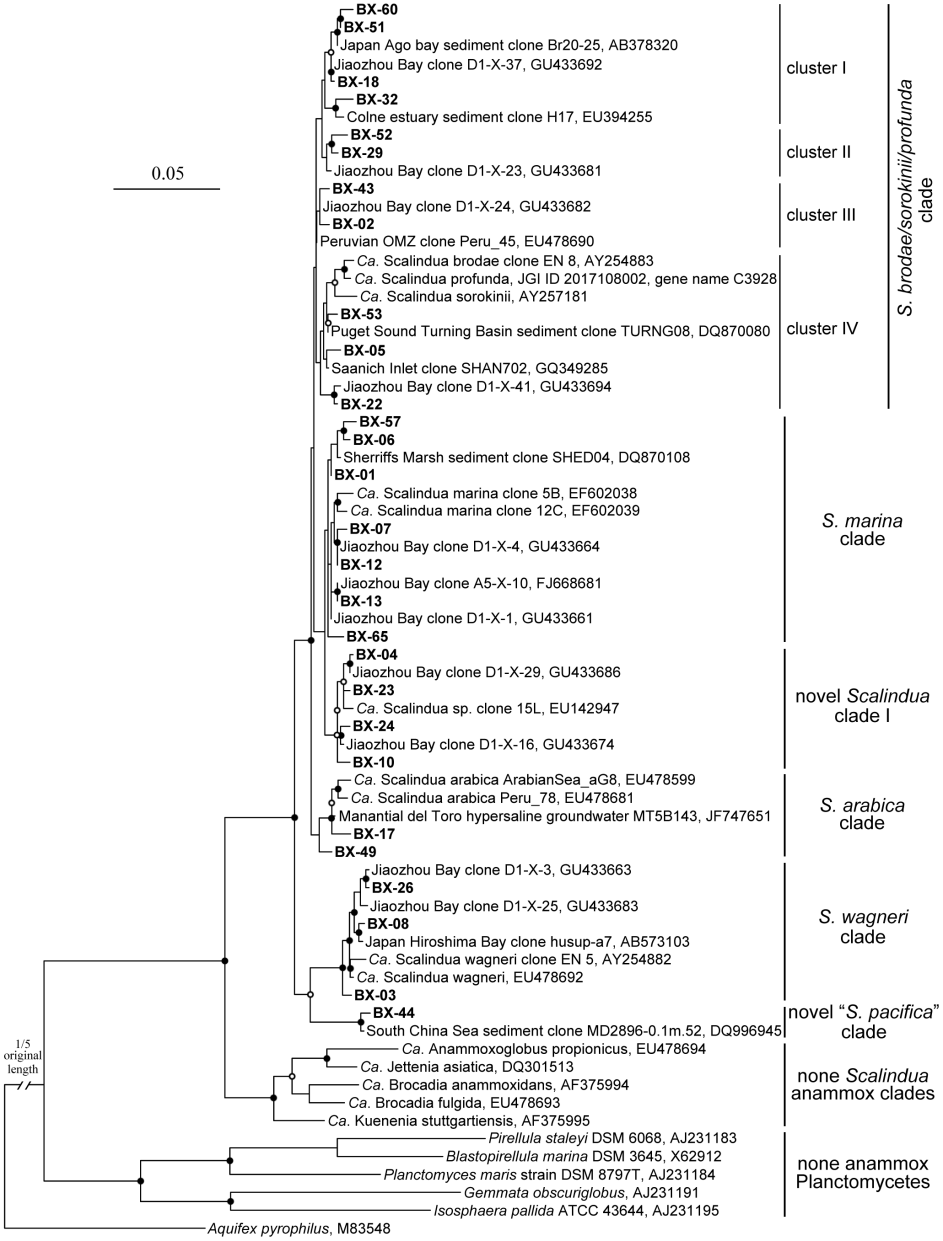
Phylogenetic analysis of representative *Ca*. Scalindua 16S rRNA gene sequences obtained from the Bohai Sea. The tree branch distances represent nucleotide substitution rate, and the scale bar represents the expected number of changes per homologous position. The *Aquifex pyrophilus* 16S rRNA gene sequence was used as outgroup. Bootstrap values (100 resamplings) higher than 70% are shown with solid circle symbols and those less than 70% but greater or equal to 50% are shown with open circle symbols on the corresponding nodes. The *Ca*. Scalindua 16S rRNA gene sequences obtained in this study are shown in bold.

**Figure 3:**
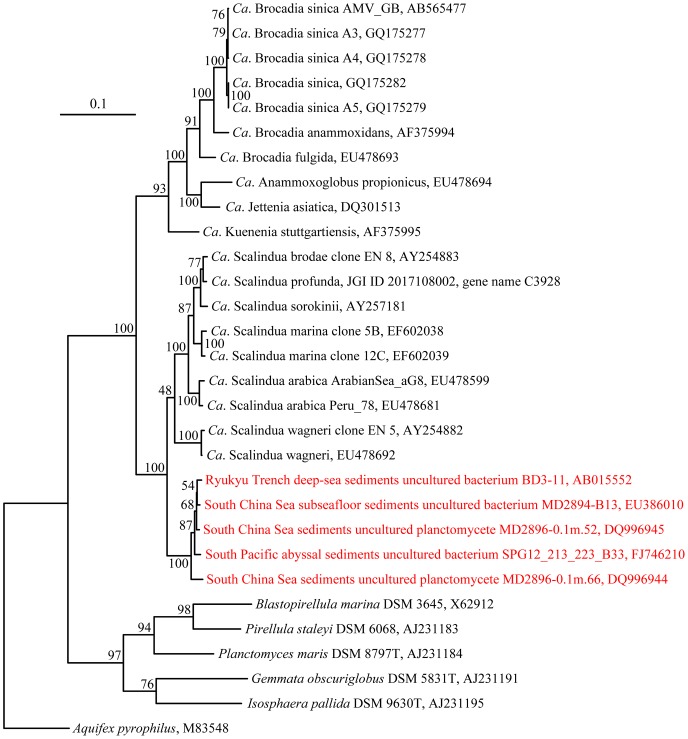
Distance neighbor-joining phylogenetic tree of nearly full-length anammox bacterial and environmental 16S rRNA gene sequences. The tree branch distances represent nucleotide substitution rate and scale bar represents expected number of changes per homologous position. *A*. *pyrophilus* 16S rRNA gene sequence was used as outgroup. Bootstrap values (100 resamplings) are shown near the corresponding nodes. Sequences shown in red form a monophyletic cluster and putatively define the new anammox bacterium candidate species, “*Ca*. Scalindua pacifica”.

The constructed phylogenetic tree using partial 16S rRNA gene sequences (∼670 bp) revealed that the Bohai Sea sediments harbored diverse *Ca*. Scalindua bacteria (Fig. 2). Six *Ca*. Scalindua clades were identified, including the *Scalindua brodae/sorokinii*/*profunda* [4], [71], [72], *S*. *marina* [73], *S*. *arabica* [23] and *S*. *wagneri* [71] clades and two novel clades. The sequences in the OTU BX-44-related novel clade shared less than 95% similarity with sequences from all the other clades, indicating that the bacteria in this clade might be a new anammox species. Two almost full-length 16S rRNA gene sequences (GenBank accessions DQ996944 and DQ996945) affiliated with this novel clade were obtained in our previous study of the South China Sea deep-sea sediments [67]. They were phylogenetically reanalyzed with other nearly full-length (∼1300 bp) 16S rRNA gene sequences of well-defined anammox bacterial candidate species (Fig. 3, S4 and S5), resulting in the novelty of the new clade being confirmed as a monophyletic 16S rRNA gene sequence cluster sharing less than 92% sequence similarity with all the well-defined anammox candidate species (Fig. 3, S4 and S5). Thus, the 16S rRNA gene sequences from this clade indeed represented a new anammox bacterium candidate species, tentatively named “*Candidatus* Scalindua pacifica” (pa.ci’fi.ca. L. fem. adj. pacifica pacific; referring to the Pacific Ocean where the organism has a wide distribution).

Bacteria affiliated with the *S*. *brodae/sorokinii*/*profunda* and *S*. *marina* clades occurred in every Bohai Sea sampling station, bacteria affiliated with the novel *Scalindua* clade I occurred in 12 stations, bacteria affiliated with the *S*. *wagneri* clade occurred in 9 stations, bacteria affiliated with the *S*. *arabica* clade occurred in 5 stations, while bacteria affiliated with the *S*. *pacifica* clade occurred in only 2 stations (B12 and B22). Most of our 16S rRNA gene sequences were affiliated with the *S*. *brodae/sorokinii*/*profunda* and *S*. *marina* clades (Fig. 2). Several subclusters were found in the *S*. *brodae/sorokinii*/*profunda* clade, indicating measurable microdiversity of the *Ca*. Scalindua bacteria. Two OTUs, BX-02 affiliated with the *S*. *brodae/sorokinii*/*profunda* clade and BX-01 with the *S*. *marina* clade, occurred in all the stations. They were also predominant, accounting for 38.1% and 40.5%, respectively, of the 1632 *Ca*. Scalindua 16S rRNA gene clones, likely representing the most abundant and prevalent *Ca*. Scalindua bacteria in the Bohai Sea sediments.

### Phylogeny of the anammox bacteria Hzo protein sequences

The obtained 151 unique *hzo* gene sequences were 72.4–99.9% identical with one another and 84.4–99.9% identical to the top-hit GenBank sequences. The deduced partial Hzo protein sequences (331 amino acid residues) were 77.3–100.0% identical with one another and 91.5–100% identical to the top-hit GenBank sequences, all originally retrieved from coastal or deep-sea sediments [20], [42], [74]. The majority (94.0%) of the top-hit GenBank sequences were retrieved from the hypernutrified Jiaozhou Bay sediments [20], strongly indicating similar anammox bacteria assemblages of these two marine environments.

**Figure 4:**
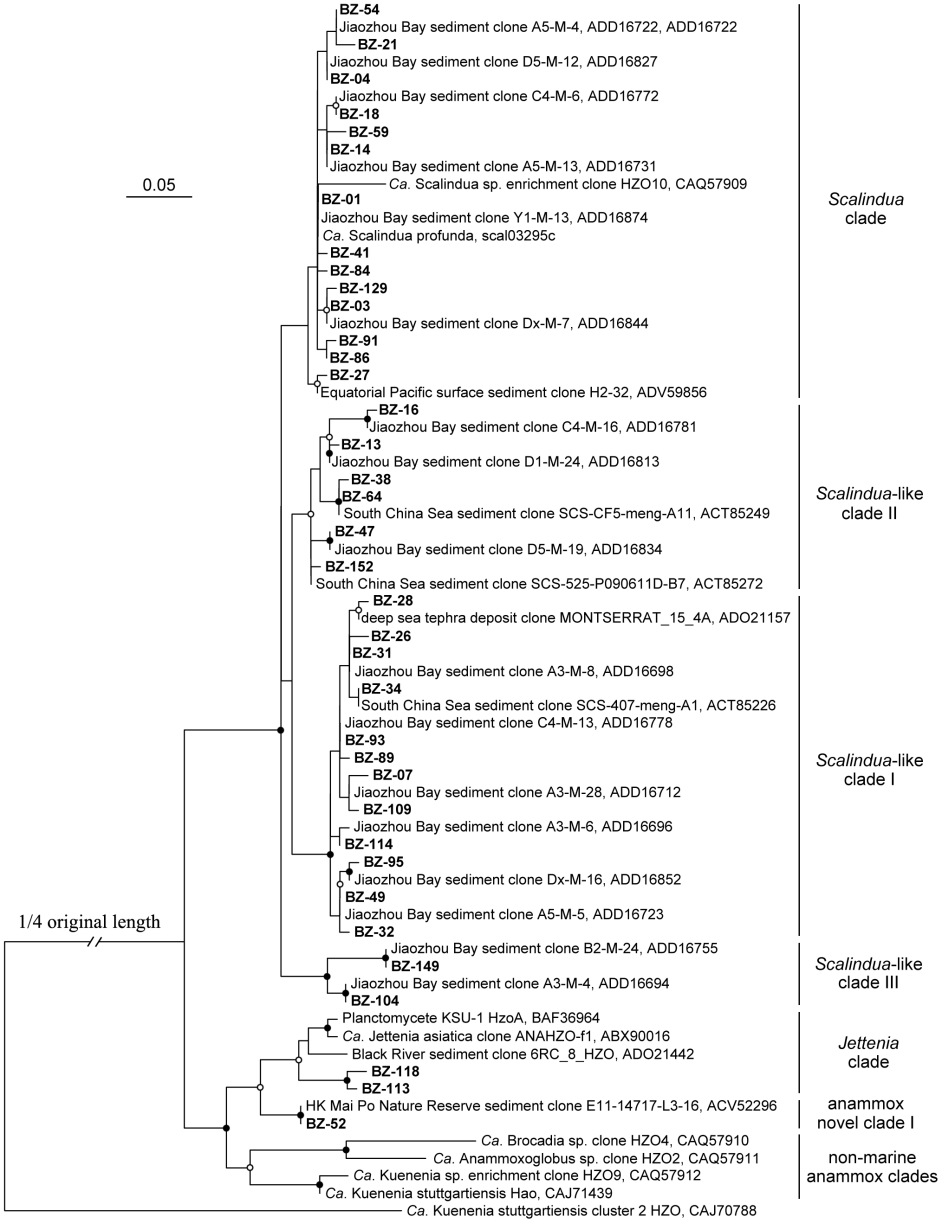
Phylogenetic analysis of representative Hzo protein sequences deduced from obtained Bohai Sea *hzo* gene sequences. The tree branch distances represent amino acid substitution rate, and the scale bar represents the expected number of changes per homologous position. The cluster 2 Hzo sequence (GenBank accession CAJ70788) was used as outgroup. Bootstrap values higher than 70% of 100 resamplings are shown with solid circle symbols, and those less than 70% but greater or equal to 50% are shown with open circle symbols on the corresponding nodes. The anammox bacterial Hzo sequences obtained in this study are shown in bold.

The Hzo phylogenetic tree reveals diverse anammox bacteria in the Bohai Sea sediments. Six distinct Hzo clades were identified (Fig. 4), including the *Scalindua* clade [19], *Scalindua*-like clade I, *Scalindua*-like clade II, *Scalindua*-like clade III, *Jettenia* clade [52] and novel anammox clade I, which were all previously found and defined in a study of the hypernutrified Jiaozhou Bay [20]. Bacteria that harbored the *Scalindua* clade and *Scalindua*-like clade I *hzo* sequences occurred in all the Bohai Sea sampling stations, while bacteria that harbored the *Scalindua*-like clade II *hzo* sequences occurred in 8 stations and bacteria that harbored the *Scalindua*-like clade III *hzo* sequences occurred in only 3 stations. Bacteria that harbored the *Jettenia* clade *hzo* sequences occurred only in station B14 and bacteria that harbored the novel anammox clade I *hzo* sequences occurred only in stations B1 and B3. Three Hzo OTUs, BZ-02, BZ-03 and BZ-79 all affiliated with the *Scalindua* clade, occurred in all the stations. They were also predominant in our clone libraries, accounting for 18.6%, 26.0% and 19.7%, respectively, of the 1490 anammox *hzo* gene clones, likely representing the most abundant and prevalent *Ca*. Scalindua bacteria in the Bohai Sea sediments.

### *Ca*. Scalindua community structure and spatial distribution

**Figure 5:**
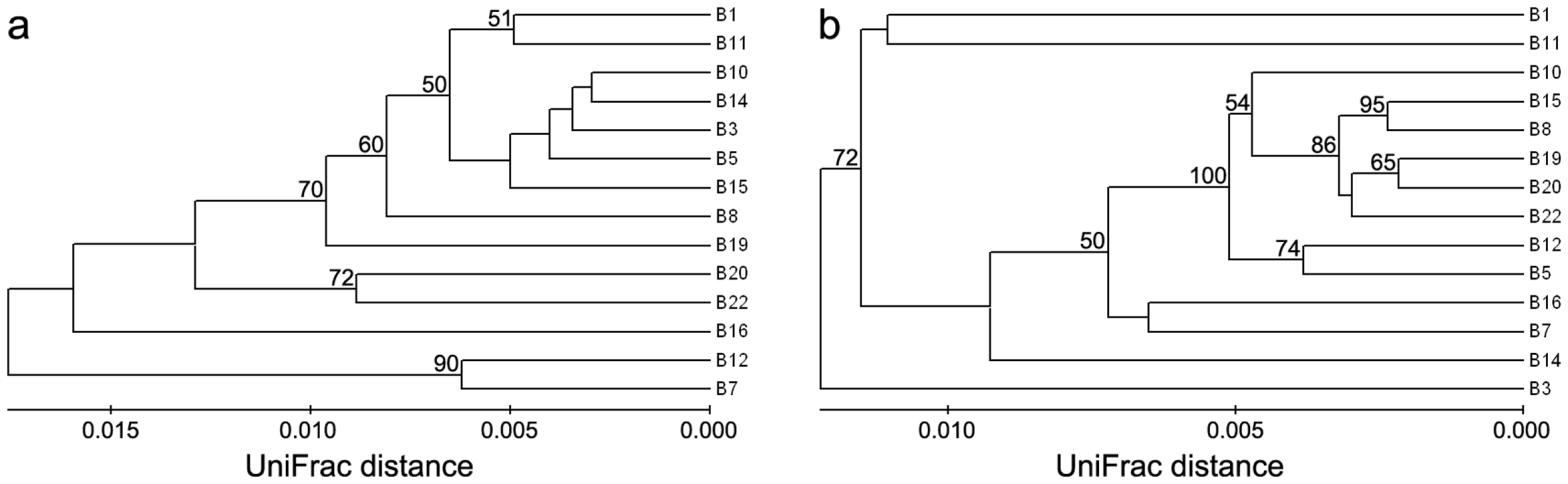
Dendrograms of hierarchical clustering analyses of the Bohai Sea sediment anammox bacterial assemblages. (a) Dendrogram of the hierarchical clustering analysis based on the *Ca*. Scalindua 16S rRNA gene sequences, and (b) dendrogram of the hierarchical clustering analysis based on the Hzo protein sequences. Both clustering dendrograms were obtained by using the Fast UniFrac normalized and weighted Jackknife Environment Clusters statistical method. The percentage supports of the classification tested with sequence jackknifing resamplings are shown near the corresponding nodes.

**Figure 6:**
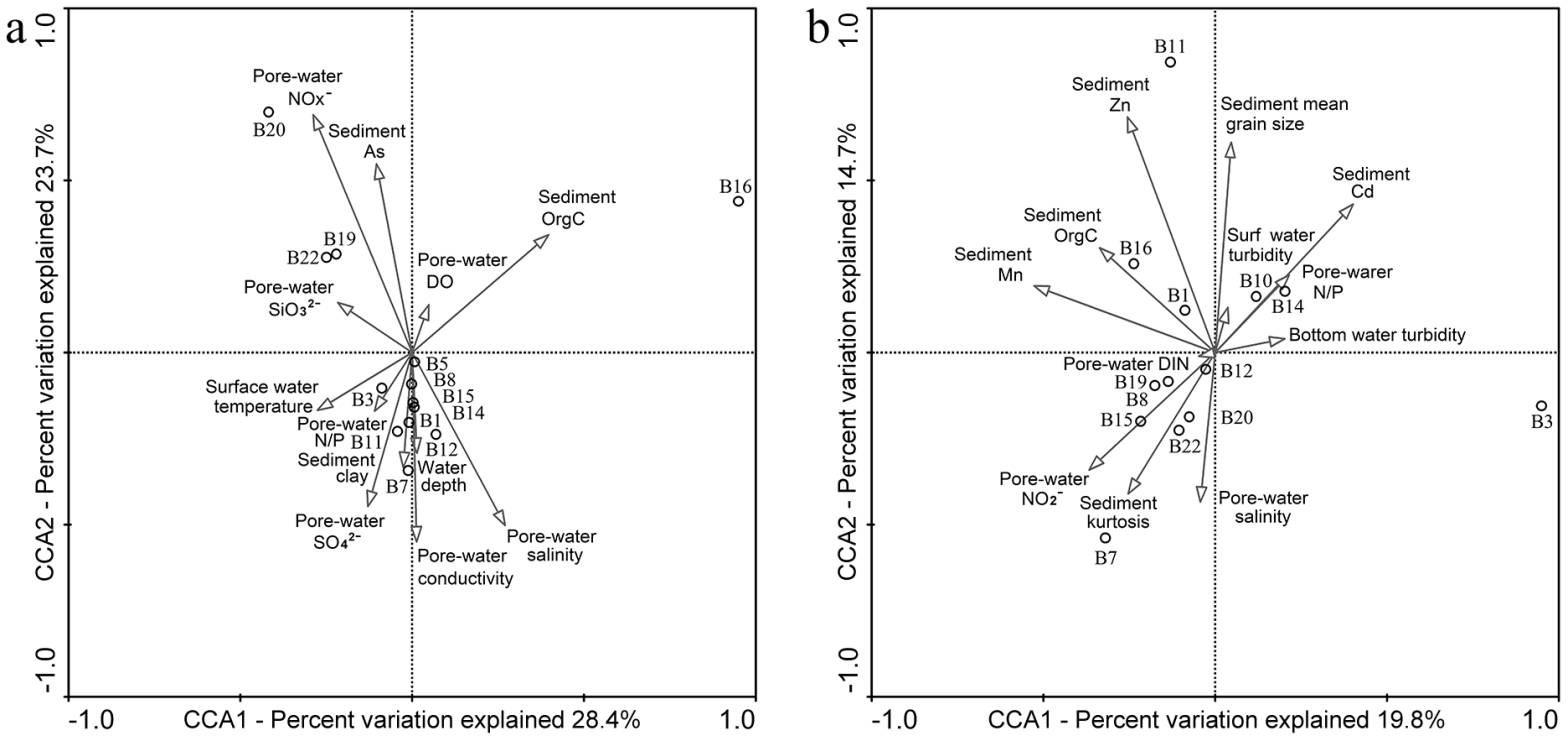
CCA ordination plots of the relationship between Bohai Sea sediment anammox bacterial assemblages and environmental factors. Only the first two principal dimensions of the CCA results were shown using the data of (a) the *Ca*. Scalindua 16S rRNA gene sequence OTUs and (b) the anammox bacteria Hzo sequence OTUs. Correlations between the Bohai Sea environmental factors and CCA axes are represented by the length and angle of arrows (environmental factor vectors). Covarying environmental variables (defined as *r*≥0.950), such as surface seawater salinity and EC25 (*r* = 0.994) and bottom seawater salinity and EC25 (*r* = 0.958), were checked to minimize collinearity in the analyses.

Environmental clustering and PCoA statistics using the obtained 16S rRNA gene sequences showed heterogeneous spatial distribution of the sediment *Ca*. Scalindua assemblages in Bohai Sea (Fig. 5a and S6a). The *Ca*. Scalindua assemblages of stations B7 and B12 were quite different from those of the other stations (Fig. 5a). The central Bohai Sea and Bohai Strait areas where stations B7 and B12 located usually have good environment quality due to efficient water exchange with the outer Yellow Sea and their distance from estuaries (Fig. 1). Environmental condition might play a key role in shaping the community structure and spatial distribution of the sediment *Ca*. Scalindua bacteria. To test this, CCA was performed (Fig. 6a). The first two CCA axes (CCA1 and CCA2) explained 51.2% of the total variance in the *Ca*. Scalindua composition and 52.1% of the cumulative variance of the *Ca*. Scalindua-environment relationship. Of all the environmental factors analyzed, sediment pore-water conductivity (*p* = 0.032) and sediment As (*p* = 0.037) were identified as the most significant environmental factors and sediment pore-water NO_x_^−^ (*p* = 0.079), SO_4_^2−^ (*p* = 0.052) and sediment OrgC (*p* = 0.094) identified as the moderately significant environmental factors in correlation with the variation of the sediment *Ca*. Scalindua community structure and spatial distribution. Collectively, these environmental factors provided 58.1% of the total CCA explanatory power, to which sediment pore-water NO_x_^−^ contributed the most (15.2%).

Correlation of the *Ca*. Scalindua assemblages with environmental factors was further analyzed with CCA using the *Ca*. Scalindua clade data identified in the phylogenetic analysis (Fig. 2). The first two CCA axes explained 82.1% of the total variance in the *Ca*. Scalindua clade composition and 82.4% of the cumulative variance of the *Ca*. Scalindua clade-environment relationship (Fig. S7). Sediment pore-water NO_x_^−^ (*p* = 0.002), sediment OrgC/OrgN (*p* = 0.032), clay content (*p* = 0.036) and kurtosis (*p* = 0.009) were identified as the most significant environmental factors and sediment Cd (*p* = 0.061), Zn (*p* = 0.072) and pore-water salinity (*p* = 0.075) identified as the moderately significant environmental factors in correlation with the variation of the sediment *Ca*. Scalindua clade composition and spatial distribution. Collectively, these environmental factors provided 91.3% of the total CCA explanatory power, to which sediment pore-water NO_x_^−^ contributed the most (43.5%). The CCA result indicated that the anammox bacteria in the *S*. *brodae/sorokinii*/*profunda* and *S*. *marina* clades had a wide spatial distribution and they might be well adapted to different environmental conditions of the Bohai Sea; bacteria in the *S*. *arabica* clade and novel *Scalindua* clade I responded positively to elevated sediment pore-water NO_x_^−^; bacteria in the *S*. *pacifica* and *S*. *wagneri* clades responded positively to elevated sediment OrgC/OrgN, kurtosis and sediment pore-water salinity; while bacteria in all the *Ca*. Scalindua clades might have very low or intermediate levels of tolerance to heavy metals Cd and Zn (Fig. S7).

### Anammox bacteria community structure and spatial distribution

Environmental clustering and PCoA statistics using the obtained Hzo sequences indicated heterogeneous distribution of the anammox bacterial communities in Bohai Sea (Fig. 5b and S6b). CCA result showed the first two CCA axes explaining 33.7% of the total variance in the anammox bacterial composition and 34.5% of the cumulative variance of the anammox bacteria-environment relationship (Fig. 6b). Sediment Cd (*p* = 0.037), Zn (*p* = 0.041) and pore-water DIN (*p* = 0.043) were identified as the most significant environmental factors in correlation with the variation of the anammox bacterial community structure and spatial distribution. Collectively, these environmental factors, with nearly equal contributions, provided 33.1% of the total CCA explanatory power.

Correlation of the anammox bacterial communities with environmental factors was further analyzed with CCA using the anammox Hzo clade data identified in the phylogenetic analysis (Fig. 4). The first two CCA axes explained 79.0% of the total variance in the anammox clade composition and 79.2% of the cumulative variance of the anammox clade-environment relationship (Fig. S8). Sediment pore-water N/P (*p* = 0.005), DIN (*p* = 0.014), sediment Cd (*p* = 0.006), Hg (*p* = 0.043), sulfide (*p* = 0.023) and kurtosis (*p* = 0.040) were identified as the most significant environmental factors and surface seawater turbidity (*p* = 0.087), sediment As (*p* = 0.081) and Zn (*p* = 0.080) identified as the moderately significant environmental factors in correlation with the variation of the sediment anammox bacterial clade composition and spatial distribution. Collectively, these environmental factors provided 92.3% of the total CCA explanatory power, to which sediment Cd and sediment pore-water N/P contributed the most (30.8% and 23.1%, respectively). The CCA result indicated that bacteria in the *Scalindua* clade had a wide distribution and they might be well adapted to different environmental conditions in the Bohai Sea; bacteria in the novel anammox clade I responded strongly to elevated sediment pore-water DIN, sediment Cd and kurtosis; bacteria in the *Scalindua*-like clade I and *Scalindua*-like clade III showed intermediate levels of responses to the above 3 environmental factors; bacteria in the *Jettenia* clade responded strongly to elevated sediment As, Hg, sediment pore-water N/P and surface seawater turbidity; and bacteria in the *Scalindua*-like clade II responded strongly to elevated sediment sulfide and Zn (Fig. S8).

### Anammox bacteria abundance

Melting curve analyses of the bacteria 16S rRNA, *Ca.* Scalindua 16S rRNA and anammox bacteria *hzo* genes confirmed that the fluorescent signals were obtained from specific qPCR products. qPCR standard curves relating threshold cycle (C_t_) to gene copy number revealed linearity (*R* ^2^ > 0.990) over six orders of magnitude of the standard gene concentrations (Table S3). The obtained high correlation coefficients and similar slopes indicated high qPCR primer hybridization and extension efficiencies (Table S3), making comparison of gene abundances reliable.

**Figure 7:**
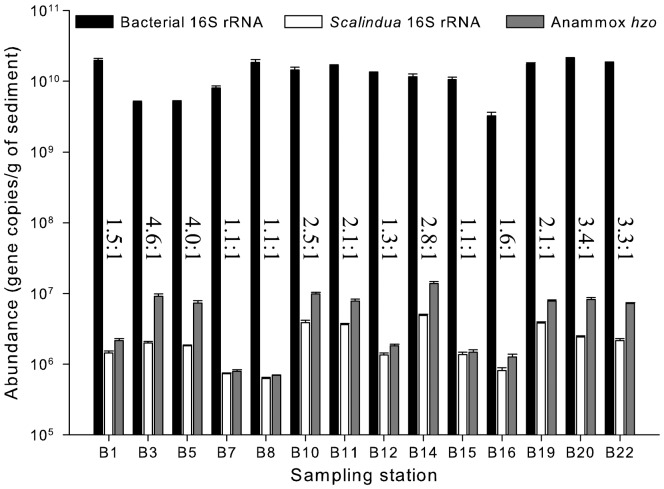
Abundances of the Bohai Sea sediment total bacteria, *Ca*. Scalindua bacteria and total anammox bacteria. These data were determined via qPCR specific to the respective target genes. The means and standard errors were calculated with three replicate qPCR measurements. The numerical values in the graph show the ratios of the total anammox bacterial *hzo* gene abundance to the *Ca*. Scalindua bacterial 16S rRNA gene abundance at each specific sampling station.

The qPCR results showed a heterogeneous spatial distribution of the sediment bacteria abundance in the Bohai Sea, where station B20 had the highest bacterial 16S rRNA gene abundance (2.10×10^10^ copies g^−1^ sediment) and station B16 had the lowest gene abundance (3.25×10^9^ copies g^−1^ sediment). The abundances of the sediment *Ca.* Scalindua and total anammox bacteria also showed heterogeneous distributions, where station B14 had the highest *Ca.* Scalindua 16S rRNA and anammox *hzo* gene abundances (4.86×10^6^ and 1.38×10^7^ copies g^−1^ sediment, respectively) while station B8 had the lowest abundances (6.22×10^5^ and 6.96×10^5^ copies g^−1^ sediment, respectively) (Fig. 7). The ratio of anammox bacteria *hzo* gene abundance to *Ca*. Scalindua 16S rRNA gene abundance ranged from 1.1∶1 (station B7) to 4.6∶1 (station B3) with an average as 2.3±1.2 in the Bohai Sea sediments (Fig. 7), close to the 2∶1 ratio in *Ca*. Kuenenia stuttgartiensis and likely also in *Ca*. Scalindua profunda and anammox bacterium strain KSU-1 [72], [75], [76].

The abundance of sediment bacteria 16S rRNA genes correlated positively with sediment porewater NO_2_^−^, surface seawater Chl-*a* and negatively with sediment pore-water salinity, surface water salinity, conductivity and density. The abundances of both sediment *Ca*. Scalindua 16S rRNA and total anammox bacteria *hzo* genes correlated positively with sediment porewater N/P, surface water temperature, turbidity and negatively with sediment porewater PO_4_^3−^. Sediment Co, Cr, Cu, Fe and Ni and certain sedimentological parameters were also significant in correlation with the abundance of the sediment *Ca*. Scalindua bacteria in the Bohai Sea (Table S2).

## Discussion

### Sediment anammox bacteria diversity

Although our samples were collected from the surface sediments (top 5-cm) of the Bohai Sea, their negative *Eh* values, relatively low DO concentrations and measurable sulfide contents (Table S1) indicate that the top 5-cm sediments also covered the suboxic and anoxic conditions, suitable for anammox bacteria and their activity. Anammox bacteria were recently found to be active over a range of DO concentrations and they may exist in broader environments than previously thought [77]. Indeed *Ca*. Scalindua bacteria, detected by both 16S rRNA and *hzo* gene PCR primers (Fig. 2, 4 and 7), were prevalent and predominant, with high microdiversity, in surface sediments of the Bohai Sea, where non-*Scalindua* anammox bacteria were also detected, by the *hzo* gene PCR primers (Fig. 4). However, the later were only found in three sampling stations, *Jettenia* clade bacteria were detected only in the Yellow River estuary station B14 and novel anammox clade I bacteria were detected only in the Liaodong Bay stations B1 and B3 that received strong river discharges such as those from the Liaohe, Daliaohe and Dalinghe rivers (Fig. 1). Our result confirmed the difference in lineage coverage of the 16S rRNA and *hzo* gene PCR primers [5], [19]–[21], [52] and also confirmed previous findings that marine non-*Scalindua* anammox bacteria occurred mainly in estuarine and coastal environments that received strong terrestrial influence [15], [20], [21], [24], [26], except the finding of similar bacteria in deep-sea hydrothermal environments [25]. Anammox bacteria in the *Anammoxoglobus*, *Brocadia*, *Jettenia* and *Kuenenia* lineages are frequently found in terrestrial, freshwater or wastewater processing systems [9], [11], [22], [78]. The source of these bacteria in marine sediments may be autochthonous or allochthonous, being residential or transported to the coastal environments via river runoffs and/or wastewater effluents [20], [79]. The presence of non-*Scalindua* anammox bacteria in deep-sea hydrothermal environments favors the first possibility [25], while the lack of anammox activity of the non-*Scalindua* anammox bacteria found in some coastal sites favors the second possibility [24]. The origin and ecofunction, if any, of the non-*Scalindua* anammox bacteria in marine environment need to be further studied.

A novel anammox bacterial 16S rRNA gene clade putatively representing a new candidate species, “*Ca*. Scalindua pacifica”, was identified via 16S rRNA gene sequence phylogenetic analyses (Fig. 2, 3, S4 and S5). Besides in the Bohai Sea, sequences affiliated with this clade have also been found in deep-sea sediments of the South China Sea, South Pacific abyssal basin and Ryukyu Trench in the Pacific Ocean [67], [80], [81], indicating that *Ca*. S. pacifica bacteria have a wide biogeographical distribution in the Pacific Ocean and they may prefer deep-sea sediment environments (Fig. 3, S4 and S5). Our CCA analysis supports this speculation that these bacteria might prefer oligotrophic marine environments (such as deep-sea sediments) as they responded to elevated sediment OrgC/OrgN (Fig. S7). The deep-sea sediment organic matter (OM) abundance and quality depend mainly on the deposition of OM produced in the photic zone of the ocean and on the complex biochemical transformations of the sinking particles occurring in water column and at seafloor [82]. As most organic nitrogen produced in the surface ocean is readily degradable, the deep-sea sediments usually have low OrgN content and quality (indicated by high OrgC/OrgN ratio). *Ca*. S. pacifica may also have very low tolerance to heavy metals such as Cd and Zn (Fig. S7). Our study putatively identifies an important sediment anammox bacterium and provides certain clues about its ecophysiology. The biogeochemical functions and genetic and biochemical mechanisms of this novel bacterium worth being further studied with metagenomic approaches.

The Hzo phylogenetic analysis has also identified several novel anammox bacterial clades (Fig. 4). However, *hzo* sequences are currently not available for many anammox bacterial candidate species. Whether these novel clades represent new anammox bacterial species cannot be solved. At present, no pure isolate of anammox bacteria can be obtained due to their very unique physiology. Metagenomic studies may help solve many puzzles about the phylogeny, ecophysiology and biogeochemical functions of the anammox bacteria in natural environment [72].

Our study identified certain different environmental responses of the *Ca*. Scalindua assemblages and the whole anammox bacterial communities in the Bohai Sea sediments (Fig. 6, S7 and S8, Table S2). The 16S rRNA gene PCR primers used only detect the anammox bacteria in the *Ca*. Scalindua lineage [5], whereas the *hzo* PCR primers putatively detect all the known anammox bacterial lineages including non-*Scalindua* species [19], [21], [52]. The *Ca*. Scalindua bacteria constitute only a component, though likely the major one [20], of the whole anammox bacterial communities in marine environment. Although the different coverage of the anammox bacterial lineages by these distinct PCR primers makes a direct parallel comparison of the 16S rRNA gene- and *hzo* gene-based results impossible, both helped decode, at different levels, and thus provide complementary views of the anammox bacterial ecological characteristics in the Bohai Sea sediments.

### Sediment anammox bacterial potential ecophysiology inferred from DNA-based analyses

Statistical analyses indicated that the diversity, community structure and abundance of the sediment anammox bacteria varied significantly in different environments of the Bohai Sea. Some clades of the anammox bacterial communities and the *Ca*. Scalindua assemblages could be found in all the Bohai Sea sampling stations, whereas other clades were only found in specific stations. CCA statistical results showed that the presence or absence of the specific clades in specific sampling stations related to specific environmental characteristics of the stations (Fig. S7 and S8). The *in situ* physical and geochemical condition, rather than localized dispersal, may play a key role in controlling the distribution of the sediment anammox bacterial assemblages in the Bohai Sea.

Diverse chemical environments, including the availability of anammox substrates (inorganic N compounds) and other reductants and oxidants, were identified to likely play important roles in shaping the sediment anammox bacterial ecophysiology in the Bohai Sea. Both NO_2_^−^ and NH_4_^+^ are the required substrates for anammox [3]. In marine environments, NO_2_^−^ is usually of very low concentration and likely the limiting resource for the anammox bacterial community and activity [20], [83]–[85]). As environmental NO_2_^−^ is usually obtained from NO_3_^−^ reduction, NO_3_^−^ or NO_x_^−^ (*i.e.*, Σ[NO_2_^−^ + NO_3_^−^]) sometimes may also be limiting for anammox [40], [86]. Indeed, the sediment pore-water NO_2_^−^, NO_3_^−^, NO_x_^−^ and NO_x_^−^/NH_4_^+^ were all found to positively influence the marine *Ca*. Scalindua bacteria diversity (Table S2), and NO_x_^−^ was found to be a key environmental factor influencing the community structure and spatial distribution of the *Ca*. Scalindua bacteria in the Bohai Sea (Fig. 6a and S7). The dependence of *Ca*. Scalindua bacteria upon the NO_2_^−^ and NO_3_^−^ availability indicates that these Bohai Sea sediment bacteria were probably active in consuming NO_2_^−^ as a substrate and catalyzing the *in situ* anammox process.

For the analyses using the Hzo sequences, no correlation was found of the sediment pore-water NO_2_^−^, NO_3_^−^ or NO_x_^−^ with the anammox bacterial diversity or community structure in the Bohai Sea. However, sediment pore-water DIN was identified as a key environmental factor influencing the overall anammox bacterial community structure and spatial distribution (Fig. 6b and S8). Sediment pore-water DIN concentrations varied significantly (from 41.32 µM to 261.48 µM) in different environments of the Bohai Sea (Table S1). In marine sediments, several other microbial N transforming processes may happen simultaneously and in close spatial proximity, especially at the oxic-anoxic interface, to compete with anammox for the availability of fixed nitrogen, such as denitrification, ammonia oxidization, nitrite oxidation, nitrate ammonification and assimilatory uptake [20], [86]-[91]. Thus, environmental DIN when its total concentration is low may become a limiting resource for anammox. This is in stark contrast to what was found in the hypernutrified Jiaozhou Bay, where the sediment pore-water total inorganic N nutrients (especially from NH_4_^+^ at 434.1–750.1 µM) are much more abundant and less variable and thus the availability of NO_2_^−^ (at 9.5–12.9 µM) becomes the limiting factor for the sediment anammox bacteria [20].

The importance of DIN to the sediment anammox microbiota is further supported by our finding that the relative availability of inorganic N and inorganic P nutrients (*i.e.*, the N/P ratio) was another key environmental factor influencing the total anammox bacterial community structure and spatial distribution (Fig. S8) and the abundance of both total anammox and *Ca*. Scalindua bacteria (Table S2). Interestingly, sediment pore-water PO_4_^3−^ showed a significantly negative influence on anammox bacterial abundance in the Bohai Sea (Table S2). PO_4_^3−^ at concentrations > 0.2 mM was found to inhibit anammox activity in batch experiments with biomass inoculated from anammox pilot plants [92]. The putatively negative impact of sediment soluble reactive P availability on anammox bacterial abundance may compound with the impact of the availability of sediment soluble reactive N. Sediment pore-water PO_4_^3−^ had the highest values in the central Bohai Sea and the lowest values in certain sampling stations of the Yellow River estuary, while sediment pore-water N/P showed a roughly opposite distribution pattern with the highest values in certain stations at the Yellow River estuary and the inner part of the Liaodong Bay and the lowest values in the stations at the central Bohai Sea and the Bohai Strait (Table S1, Fig. 1). Our study indicates that the regime of environmental nutrients may support distinctly different anammox bacterial community structure and abundance in different sediment environments of the Bohai Sea.

Surprisingly, sediment pore-water SO_4_^2−^ and sediment sulfide content were also identified as key environmental factors influencing the community structure and spatial distribution of the sediment anammox bacteria (Fig. 6a and S8). S cycle (sulfate reduction and sulfide oxidation) plays important roles in providing extra supply of NH_4_^+^ and NO_2_^−^ and thus influencing the anammox process in the oxygen minimum zone off the Chilean coast [93]. This type of active *in situ* coupling between the S and N cycles may also exist in marine sediments [94], [95]. An anammox bacterium named “*Anammoxoglobus sulfate*” may even possess the potential of anaerobic ammonium oxidation via sulfate reduction [96]. The contribution of inorganic S chemicals to the marine anammox process deserves to be further investigated.

Sediment OrgC was found as a key environmental factor influencing the community structure and spatial distribution of the marine *Ca*. Scalindua bacteria and the overall anammox bacterial biodiversity (Fig. 6a, Table S2). The Yellow River estuary sediments usually had the highest OrgC (Table 1, Fig. 1), likely supplied by terrestrial input via river discharge [97]. A previous study found that the total availability of sediment OrgC, rather than its quality, coupled to a supply of NO_3_^−^, maintains the anammox reaction potential in estuarine sediments [40]. However, the current study identified sediment OrgC/OrgN as another key environmental factor influencing the sediment anammox bacterial community structure and distribution (Fig. S7). Consistent to this finding, sediment OrgC/OrgN was also identified as a key environmental factor influencing the sediment anammox bacterial community structure and distribution in Jiaozhou Bay [20]. OrgC/OrgN may represent the biodegradability and utilizability of sediment OM with high OrgC/OrgN usually standing for old and recalcitrant OM and low OrgC/OrgN for fresh and labile OM. Thus, the quality of sediment OM may play a role in shaping the sediment anammox microbiota. Marine Ca. *Scalindua* may utilize organic acids as a supplementary carbon source and take up amino acids, oligopeptides and other small organic compounds for alternative sources of NH_4_^+^ and/or energy production [72], [98]. Degraded proteins and other biogenic organic materials, likely originating from sinking and mineralized OM from seawater euphotic zone bioproduction, may be used directly by sediment *Ca*. Scalindua bacteria [72]. The influence of sediment OrgC and OrgC/OrgN on the community structure and spatial distribution of the marine *Ca.* Scalindua bacteria supports their novel ecophysiology identified in previous studies [72], [98].

Metal-containing enzymes play critical roles in anammox bacteria biochemistry and physiology. Under cultivated conditions, marine *Ca*. S. profunda Cu ABC transport-encoding genes are highly expressed, indicating the high requirement of Cu for this bacterium [72]. *Ca*. S. profunda was found to harbor and express the genes for Fe^3+^ reduction, and thus marine *Ca*. Scalindua bacteria may use iron oxides as terminal electron acceptors, in addition to NO_3_^−^ and NO_2_^−^, for anammox or alternative energy production [72], [98]. To support the important roles of metals in marine anammox bacterial ecophysiology, both sediment Cu and Fe contents were found to positively influence the abundance of both the *Ca*. Scalindua and the total anammox bacteria in the Bohai Sea sediments (Table S2).

The strong influence of the availability of N nutrients on the anammox microbiota indicates that these sediment bacteria were probably active and catalyzing the anammox process in the Bohai Sea. Indeed a recent study reported detectable anammox activities of the estuarine and coastal sediment anammox bacterial communities in the Bohai Sea [99]. Furthermore, the potentials of OrgC, OrgN, SO_4_^2−^, sulfide and metals in participating, directly or indirectly, the anammox process may release the strict dependence of the anammox bacteria upon the direct availability of inorganic N nutrients such as NO_2_^−^, NO_3_^−^ and NH_4_^+^, which may be limiting in certain marine environments. Our study provides a support to the versatile lifestyle of the marine anammox bacteria revealed by recent enrichment, metagenomic, metatranscriptomic and metaproteomic analyses [72], [98].

### Responses of anammox microbiota to coastal environmental variations and perturbations

Bohai Sea is a semi-enclosed water body with numerous discharging rivers surrounding the coast (Fig. 1). Strong river influence is a salient factor that shapes the geochemistry, environment and ecosystem of the Bohai Sea. Our results indicate that river inputs and the associated environmental contaminants had significant influences on the sediment anammox bacterial diversity, community structure, spatial distribution and abundance.

The Bohai Sea coastal areas were found to be contaminated by various heavy metals [30]–[32], [36]. In this study, sediment heavy metal contents of As, Cd, Mn and Pb were found to be significant in constraining the anammox bacterial diversity (Table S2). The highest concentrations of sediment As and Mn are usually found at the Yellow River estuary and the highest concentrations of sediment Cd and Pb are usually found in the inner part of the Liaodong Bay that received discharges from several rivers (Table S1, Fig. 1), indicating likely anthropogenic sources of the elevated heavy metal concentrations in the Bohai Sea. Sediment As, Cd, Hg and Zn were identified as key environmental factors influencing the community structure and spatial distribution of the marine anammox bacteria (Fig. 6a, 6b, S7 and S8), and sediment Co, Cr, Cu and Ni were found to significantly influence the marine *Ca*. Scalindua abundance in the Bohai Sea (Table S2). Although the heavy metal concentrations were not very high in our sediment samples, likely due to the distance of our sampling sites from the shoreline and thus from the contamination sources, our data indicate possible riverine inputs and their influence on the sediment anammox bacteria in the Bohai Sea.

Several sedimentological parameters were also found as key environmental factors influencing the community structure, distribution and abundance of the sediment anammox bacteria in the Bohai Sea (Table S2, Fig. S7 and S8). Similar results were obtained previously in Jiaozhou Bay [20]. Sedimentological status may depend on sediment source material supplies such as those from rivers and on *in situ* hydrological dynamics such as currents, tides, waves, upwelling, lateral transport, water mixing and the intensity and duration of these activities, which strongly influence the transportation rate of various materials at the seawater-sediment interface and inside the sediments and thus the status and dynamics of sediment composition, OM content, DO concentration, redox state, composition and abundance of nutrients, and composition and abundance of environmental contaminants [45]. Therefore, the sedimentological condition may play important roles in shaping the sediment anammox microbiota in marine environment [20].

In summary, the Bohai Sea sediments harbored diverse and some novel anammox bacteria, with a new candidate species, *Ca*. Scalindua pacifica, being tentatively identified. The importance of inorganic N nutrients and many other environmental factors to the anammox microbiota suggests that these bacteria were active in the sediment N transforming process and maintained a versatile life style well adapted to the varying environmental conditions of the Bohai Sea.

## Supporting Information

Figure S1 **Rarefaction curves of the constructed gene clone libraries using the Bohai Sea sediment samples.** (a) The rarefaction curve of the *Ca*. Scalindua 16S rRNA gene sequence OTUs and (b) the rarefaction curve of the anammox bacterial Hzo protein sequence OTUs.
(TIF)

Figure S2 **Clustering dendrograms showing the similarity of the Bohai Sea duplicate subcore sediment anammox bacteria assemblages.** (a) Hierarchical clustering analysis using the *Ca*. Scalindua 16S rRNA gene sequences and (b) using the anammox bacteria Hzo protein sequences. The two *Ca*. Scalindua 16S rRNA gene clone libraries of the B22-I and B22-II sediment subcore samples of the B22 station are grouped together in diagram (a) and the two anammox bacteria *hzo* gene clone libraries of the B22-I and B22-II sediment subcore samples of the B22 station are grouped together in diagram (b), indicating high similarity of the duplicate subcore sediment samples about the clone libraries of the respective genes at station B22.
(TIF)

Figure S3 **PCoA ordination diagrams showing the Bohai Sea bacterial assemblage similarity of duplicate subcore sediment samples.** (a) The ordination diagram produced with weighted and normalized Fast UniFrac PCoA by using the *Ca*. Scalindua 16S rRNA gene sequences and (b) by using the anammox bacteria Hzo protein sequences. The two *Ca*. Scalindua 16S rRNA gene clone libraries of the B22-I and B22-II sediment subcore samples of the B22 station are located very closely to each other in diagram (a) and the two anammox bacteria *hzo* gene clone libraries of the B22-I and B22-II sediment subcore samples of the B22 station are located very closely to each other in diagram (b), indicating high similarity of the duplicate subcore sediment samples about the clone libraries of the respective genes at station B22.
(TIF)

Figure S4 **Maximum likelihood phylogenetic tree of nearly full-length anammox bacterial and environmental 16S rRNA gene sequences.** The tree branch distances represent nucleotide substitution rate and scale bar represents expected number of changes per homologous position. *A*. *pyrophilus* 16S rRNA gene sequence was used as outgroup. Bootstrap values (100 resamplings) are shown near the corresponding nodes. Sequences shown in red form a monophyletic cluster and putatively define the new anammox bacterium candidate species, “*Ca*. Scalindua pacifica”.
(TIF)

Figure S5 **Parsimony phylogenetic tree of nearly full-length anammox bacterial and environmental 16S rRNA gene sequences.** The tree branch distances represent nucleotide substitution rate and scale bar represents expected number of changes per homologous position. *A*. *pyrophilus* 16S rRNA gene sequence was used as outgroup. Bootstrap values (100 resamplings) are shown near the corresponding nodes. Sequences shown in red form a monophyletic cluster and putatively define the new anammox bacterium candidate species, “*Ca*. Scalindua pacifica”.
(TIF)

Figure S6 **Ordination diagrams of Fast UniFrac PCoA analyses of the Bohai Sea sediment anammox bacterial assemblages.** (a) The PCoA ordination diagram produced by using the *Ca*. Scalindua 16S rRNA gene sequences and (b) by using the anammox bacteria Hzo protein sequences. Shown are the plots of the first two principal coordinate axes (P1 and P2) of the weighted and normalized PCoA and the distribution of (a) the *Ca*. Scalindua 16S rRNA gene-typic assemblages and (b) the anammox bacterial Hzo protein-typic assemblages (designated with the sampling station names) in response to these axes.
(TIF)

Figure S7 **CCA ordination plot showing the relationship between the sediment** ***Ca*** **. Scalindua assemblages and environmental factors**. This plot shows the first two principal dimensions of the CCA that was conducted by using the data of the *Ca*. Scalindua 16S rRNA gene sequence clades defined in **Figure 2** of this study. Abbreviations: *Sa*, the *Scalindua arabica* clade; *Sm*, the *S. marina* clade; *Ss*, the *S. brodae/sorokinii*/*profunda* clade; *Spa*, the *S. pacifica* clade; *SnI*, the novel *Scalindua* clade I; *Sw*, the *S. wagneri* clade.
(TIF)

Figure S8 **CCA ordination plot showing the relationship between the sediment anammox bacterial assemblages and environmental factors**. This plot shows the first two principal dimensions of the CCA that was conducted by using the data of the Hzo sequence clades defined in **Figure 4** of this study. Abbreviations: AI, the novel anammox clade I; J, the *Jettenia* clade; S, the *Scalindua* clade; SI, the *Scalindua*-like clade I; SII, the *Scalindua*-like clade II; SIII, the *Scalindua*-like clade III.
(TIF)

Table S1 Measurements of *in situ* environmental parameters of the 14 sampling stations in the Bohai Sea.
(PDF)

Table S2 Correlation analyses of biodiversity and abundance of key microbial groups with Bohai Sea environmental factors.
(PDF)

Table S3 Efficiency and sensitivity of individual qPCR standard curve determined via linearized plasmid DNA.
(PDF)
