# Supplementary figures and images for: Molecular Detection of Candidatus Scalindua pacifica and Environmental Responses of Sediment Anammox Bacterial Community in the Bohai Sea, China
Source: PLoS One. 2013 Apr 8;8(4):e61330. doi: 10.1371/journal.pone.0061330 (PMC3620062; doi:10.1371/journal.pone.0061330)

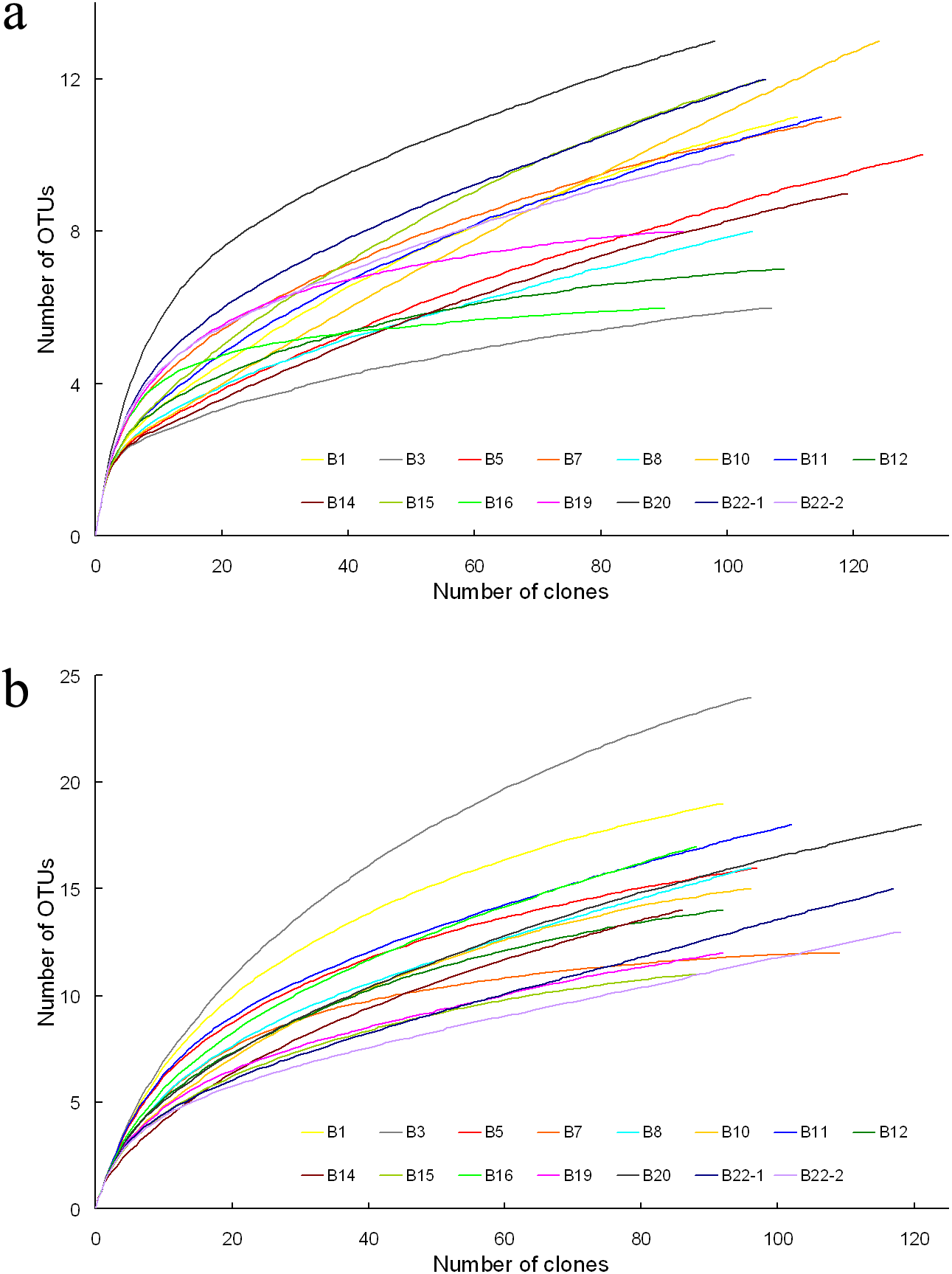

Supplement: Figure S1 — Rarefaction curves of the constructed gene clone libraries using the Bohai Sea sediment samples. (a) The rarefaction curve of the Ca. Scalindua 16S rRNA gene sequence OTUs and (b) the rarefaction curve of the anammox bacterial Hzo protein sequence OTUs. (TIF) [file pone.0061330.s001.tif]

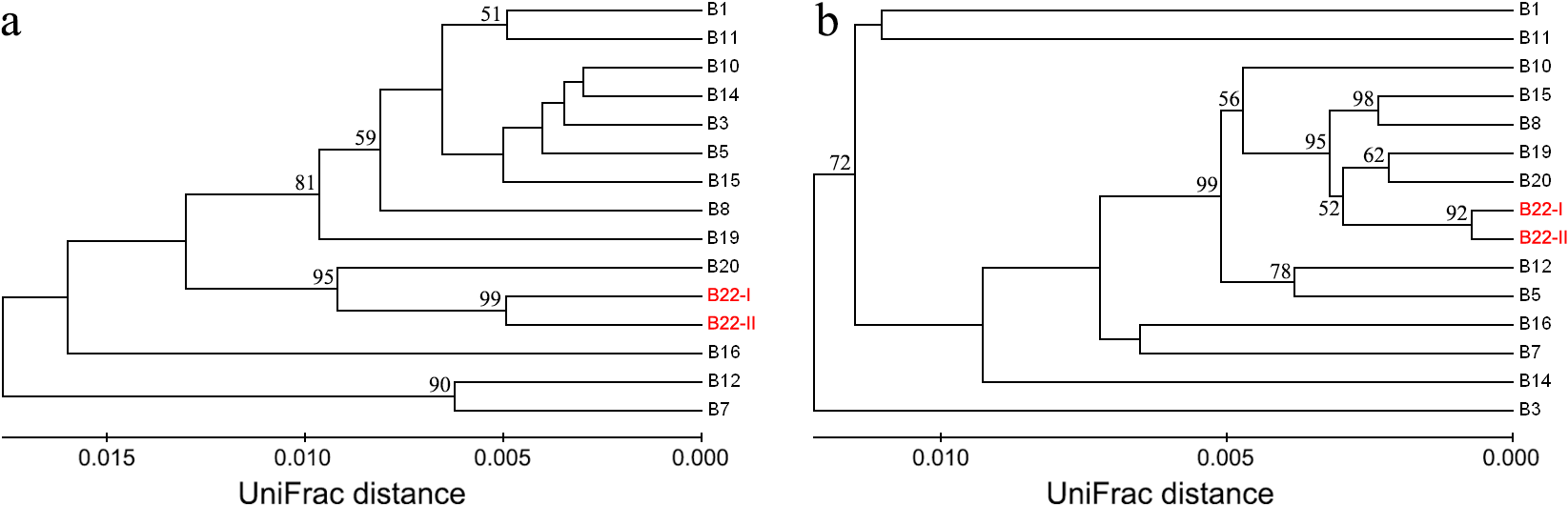

Supplement: Figure S2 — Clustering dendrograms showing the similarity of the Bohai Sea duplicate subcore sediment anammox bacteria assemblages. (a) Hierarchical clustering analysis using the Ca. Scalindua 16S rRNA gene sequences and (b) using the anammox bacteria Hzo protein sequences. The two Ca. Scalindua 16S rRNA gene clone libraries of the B22-I and B22-II sediment subcore samples of the B22 station are grouped together in diagram (a) and the two anammox bacteria hzo gene clone libraries of the B22-I and B22-II sediment subcore samples of the B22 station are grouped together in diagram (b), indicating high similarity of the duplicate subcore sediment samples about the clone libraries of the respective genes at station B22. (TIF) [file pone.0061330.s002.tif]

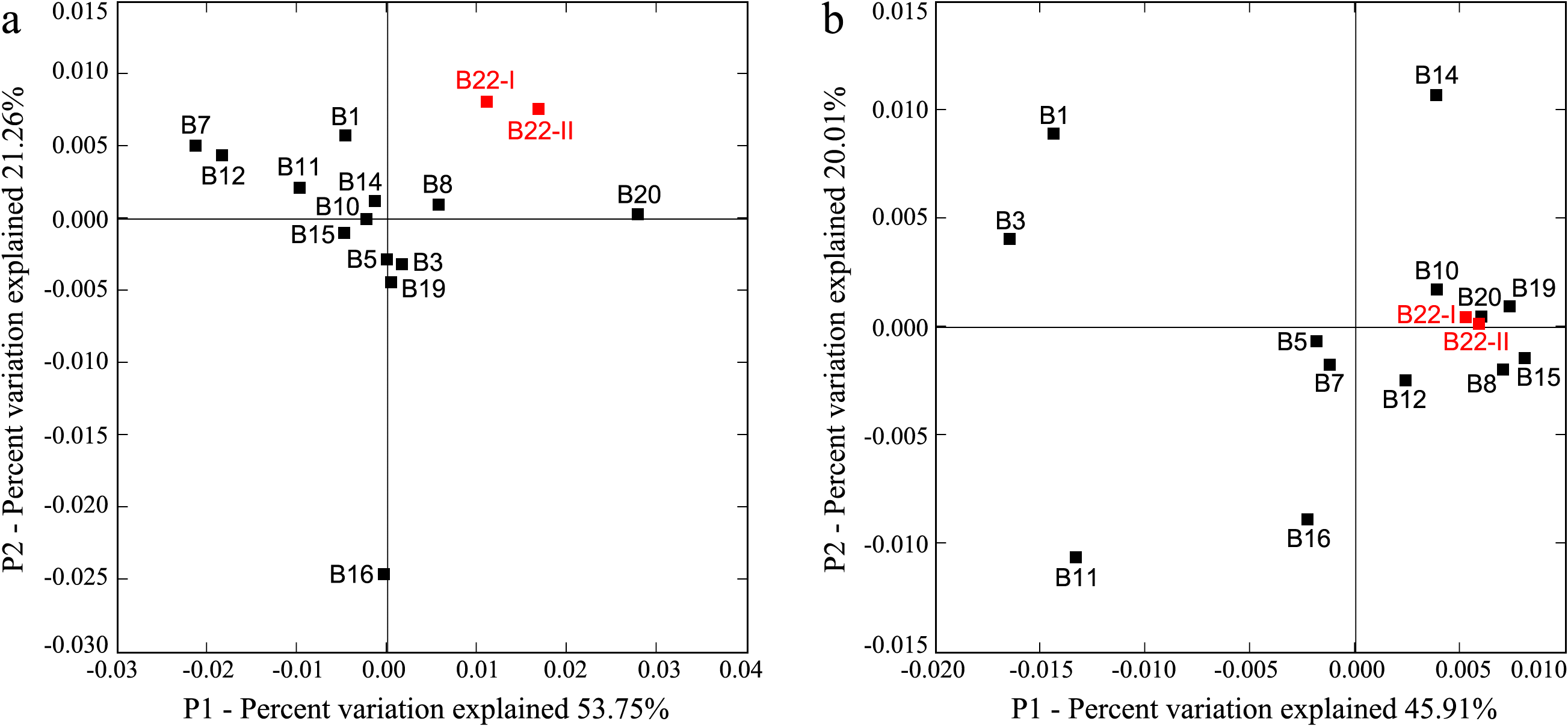

Supplement: Figure S3 — PCoA ordination diagrams showing the Bohai Sea bacterial assemblage similarity of duplicate subcore sediment samples. (a) The ordination diagram produced with weighted and normalized Fast UniFrac PCoA by using the Ca. Scalindua 16S rRNA gene sequences and (b) by using the anammox bacteria Hzo protein sequences. The two Ca. Scalindua 16S rRNA gene clone libraries of the B22-I and B22-II sediment subcore samples of the B22 station are located very closely to each other in diagram (a) and the two anammox bacteria hzo gene clone libraries of the B22-I and B22-II sediment subcore samples of the B22 station are located very closely to each other in diagram (b), indicating high similarity of the duplicate subcore sediment samples about the clone libraries of the respective genes at station B22. (TIF) [file pone.0061330.s003.tif]

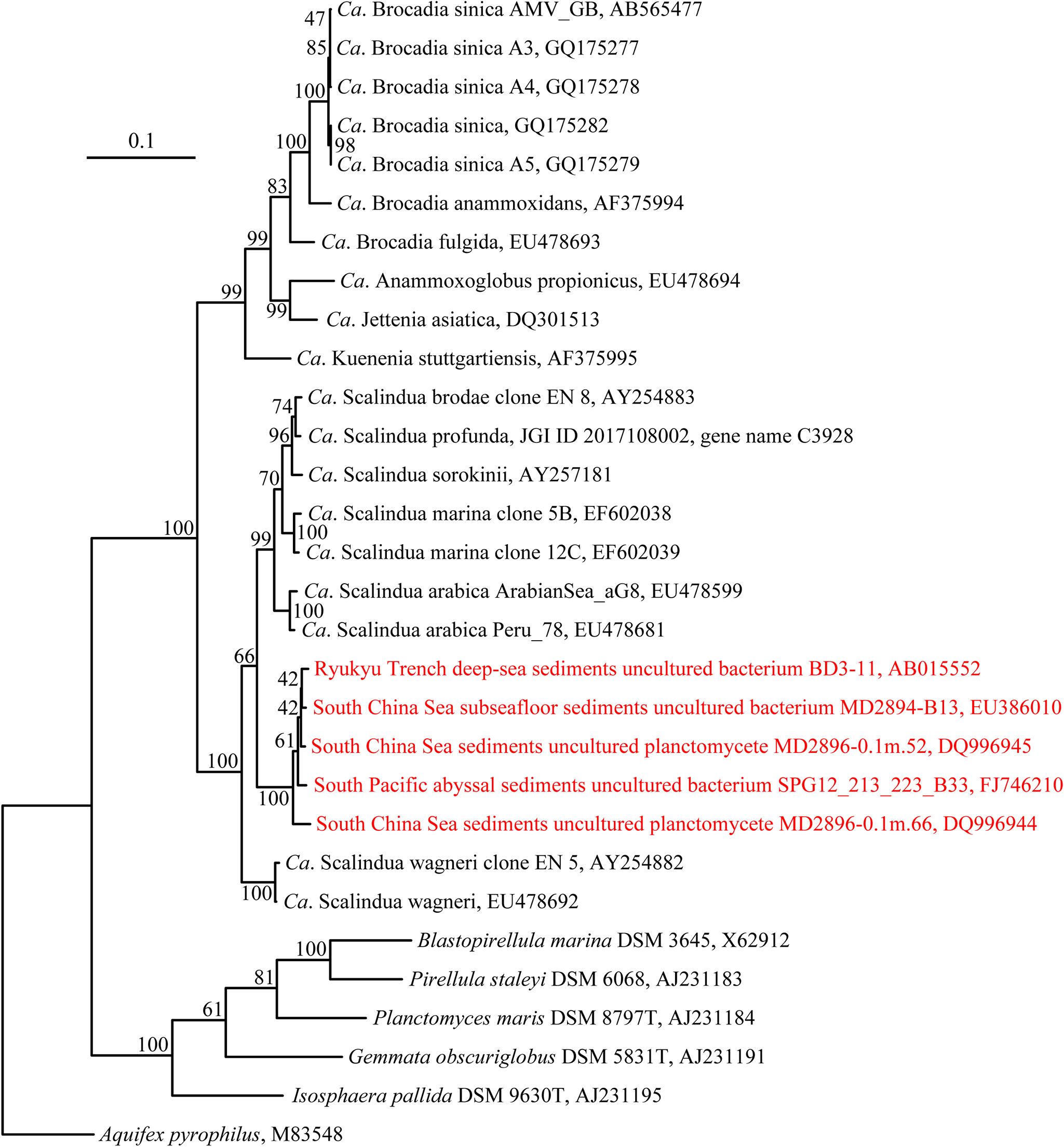

Supplement: Figure S4 — Maximum likelihood phylogenetic tree of nearly full-length anammox bacterial and environmental 16S rRNA gene sequences. The tree branch distances represent nucleotide substitution rate and scale bar represents expected number of changes per homologous position. A. pyrophilus 16S rRNA gene sequence was used as outgroup. Bootstrap values (100 resamplings) are shown near the corresponding nodes. Sequences shown in red form a monophyletic cluster and putatively define the new anammox bacterium candidate species, “Ca. Scalindua pacifica”. (TIF) [file pone.0061330.s004.tif]

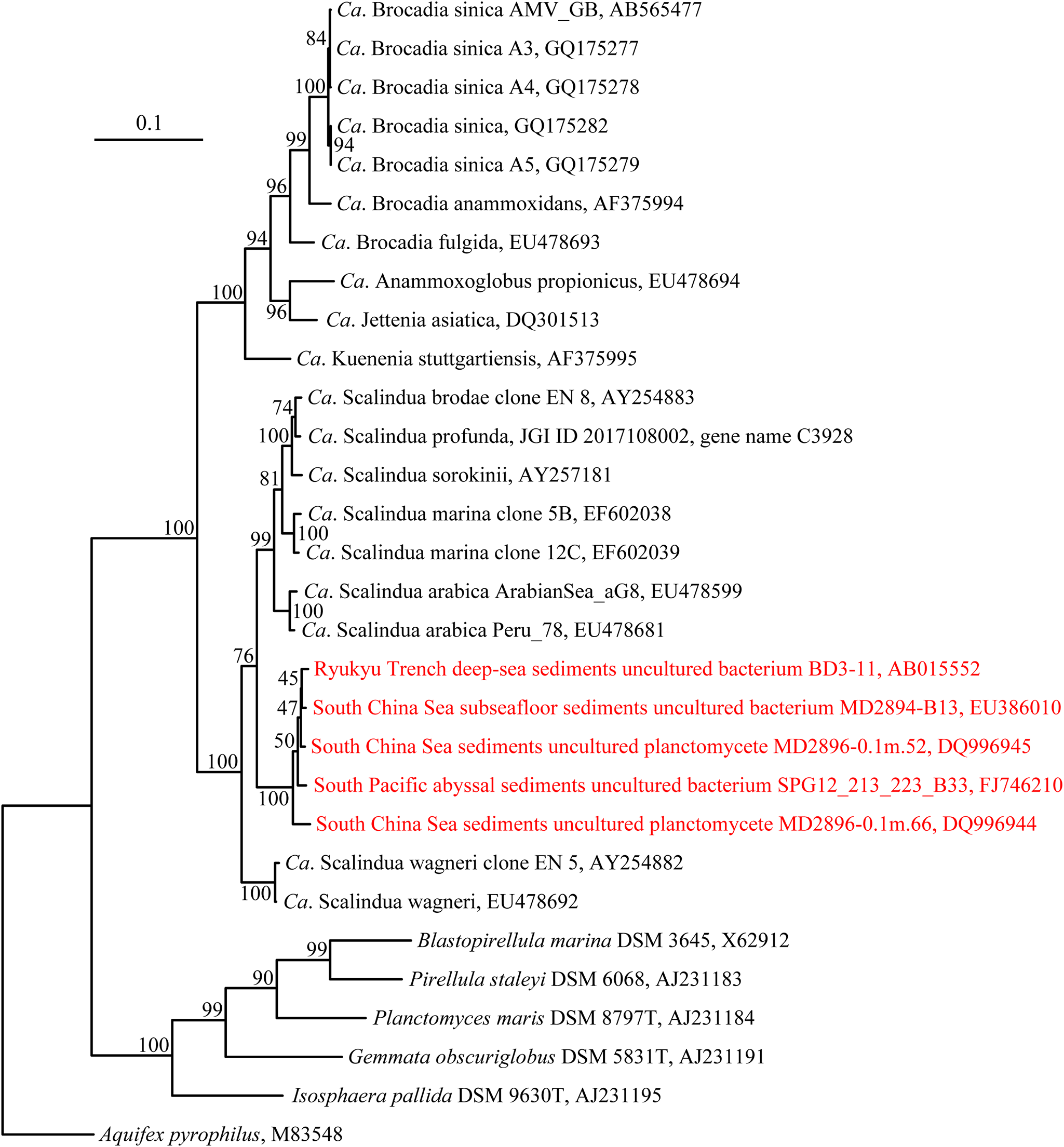

Supplement: Figure S5 — Parsimony phylogenetic tree of nearly full-length anammox bacterial and environmental 16S rRNA gene sequences. The tree branch distances represent nucleotide substitution rate and scale bar represents expected number of changes per homologous position. A. pyrophilus 16S rRNA gene sequence was used as outgroup. Bootstrap values (100 resamplings) are shown near the corresponding nodes. Sequences shown in red form a monophyletic cluster and putatively define the new anammox bacterium candidate species, “Ca. Scalindua pacifica”. (TIF) [file pone.0061330.s005.tif]

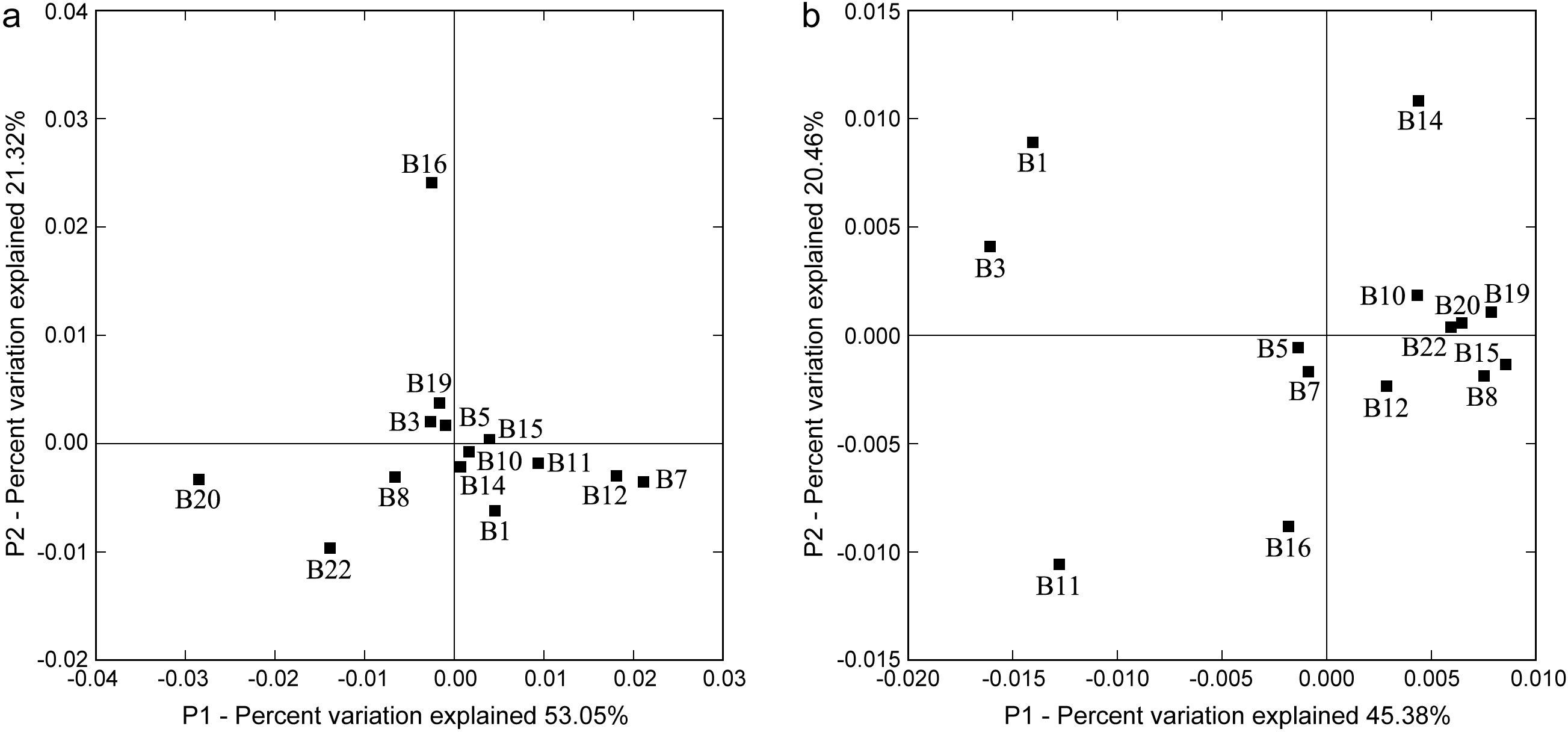

Supplement: Figure S6 — Ordination diagrams of Fast UniFrac PCoA analyses of the Bohai Sea sediment anammox bacterial assemblages. (a) The PCoA ordination diagram produced by using the Ca. Scalindua 16S rRNA gene sequences and (b) by using the anammox bacteria Hzo protein sequences. Shown are the plots of the first two principal coordinate axes (P1 and P2) of the weighted and normalized PCoA and the distribution of (a) the Ca. Scalindua 16S rRNA gene-typic assemblages and (b) the anammox bacterial Hzo protein-typic assemblages (designated with the sampling station names) in response to these axes. (TIF) [file pone.0061330.s006.tif]

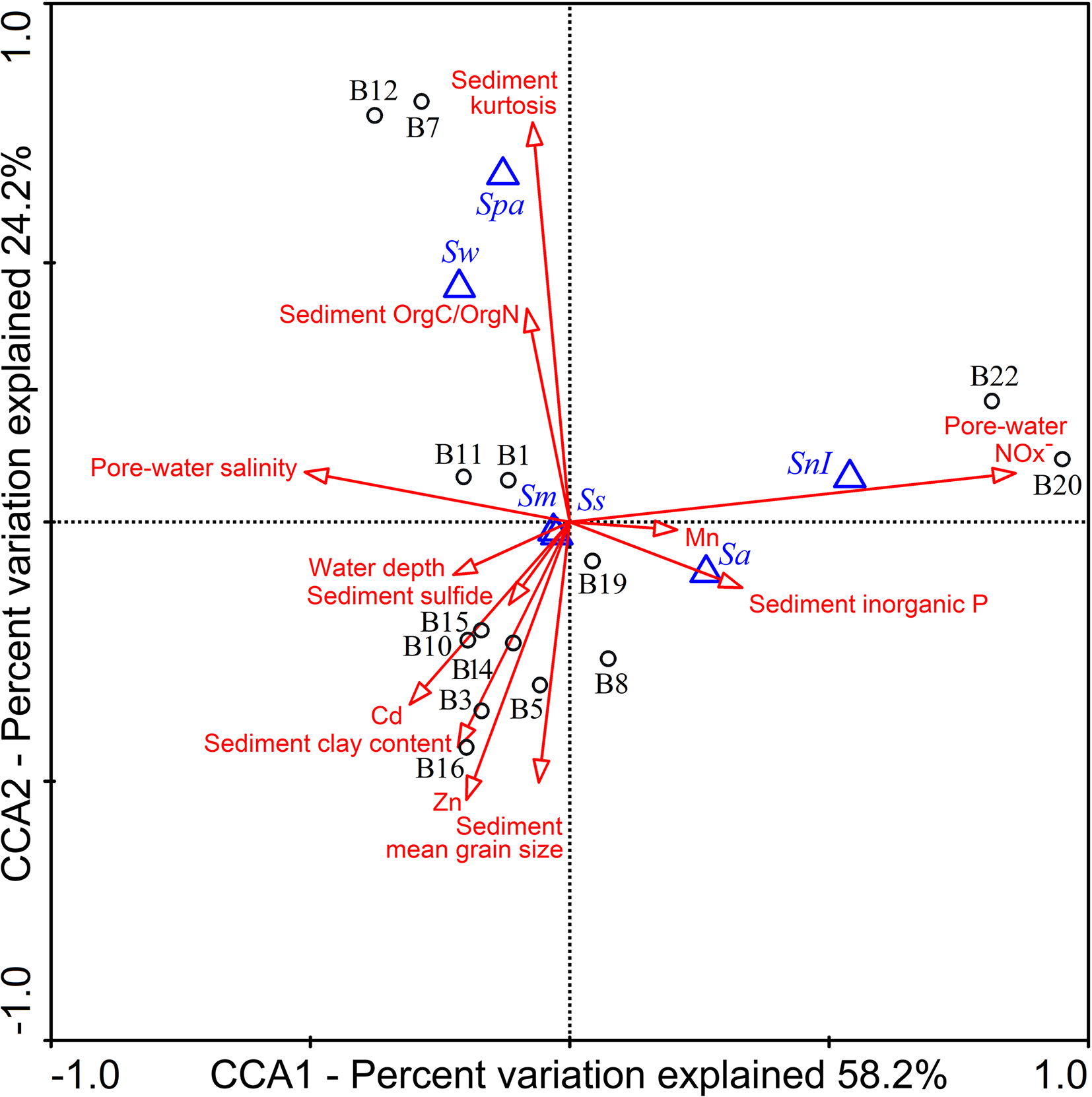

Supplement: Figure S7 — CCA ordination plot showing the relationship between the sediment Ca . Scalindua assemblages and environmental factors. This plot shows the first two principal dimensions of the CCA that was conducted by using the data of the Ca. Scalindua 16S rRNA gene sequence clades defined in Figure 2 of this study. Abbreviations: Sa, the Scalindua arabica clade; Sm, the S. marina clade; Ss, the S. brodae/sorokinii/profunda clade; Spa, the S. pacifica clade; SnI, the novel Scalindua clade I; Sw, the S. wagneri clade. (TIF) [file pone.0061330.s007.tif]

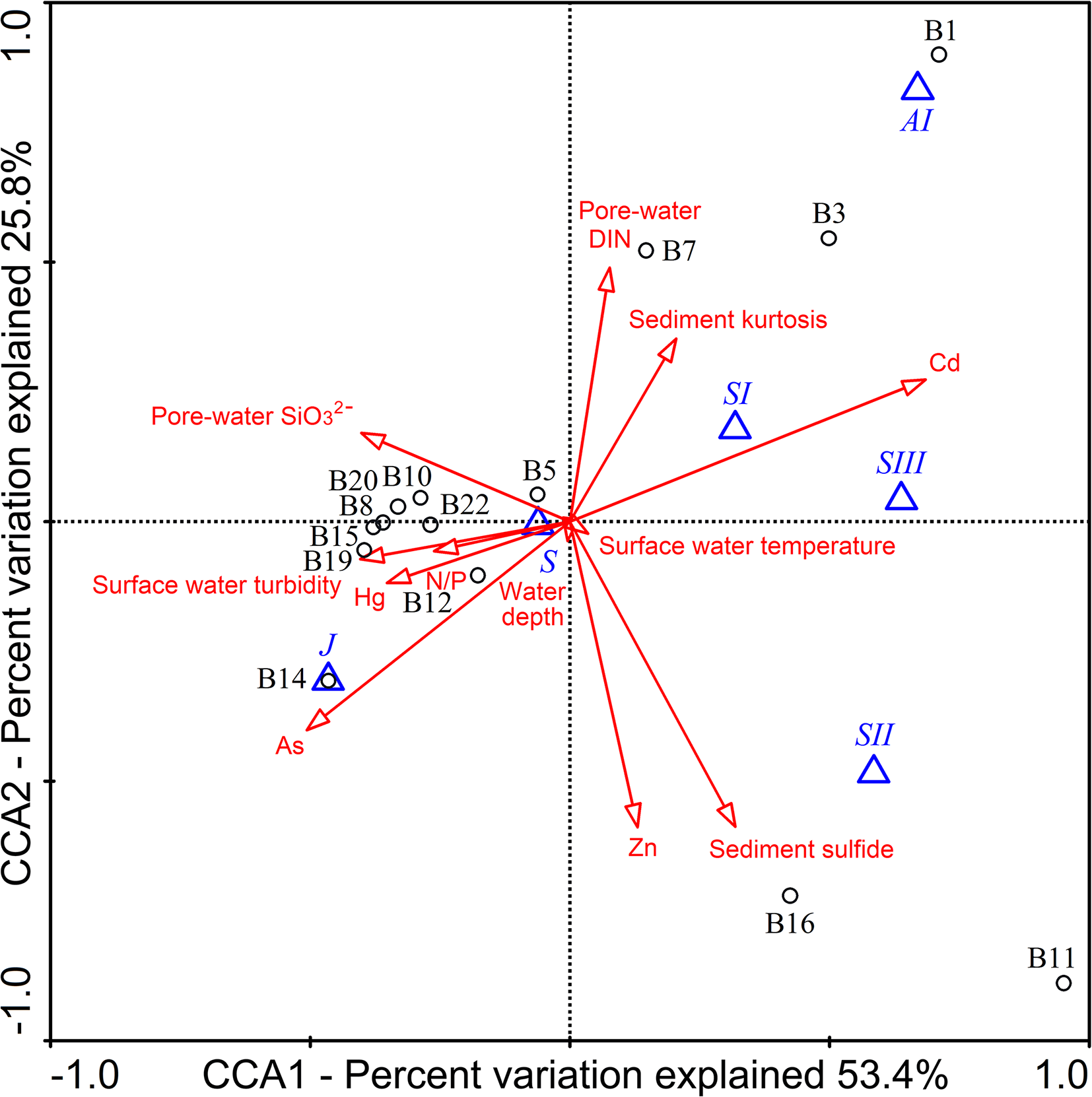

Supplement: Figure S8 — CCA ordination plot showing the relationship between the sediment anammox bacterial assemblages and environmental factors. This plot shows the first two principal dimensions of the CCA that was conducted by using the data of the Hzo sequence clades defined in Figure 4 of this study. Abbreviations: AI, the novel anammox clade I; J, the Jettenia clade; S, the Scalindua clade; SI, the Scalindua-like clade I; SII, the Scalindua-like clade II; SIII, the Scalindua-like clade III. (TIF) [file pone.0061330.s008.tif]
