# Supplementary material for: Molecular Detection of Candidatus Scalindua pacifica and Environmental Responses of Sediment Anammox Bacterial Community in the Bohai Sea, China
Source: PLoS One. 2013 Apr 8;8(4):e61330. doi: 10.1371/journal.pone.0061330 (PMC3620062; doi:10.1371/journal.pone.0061330)
Supplement: Table S1 — Measurements of in situ environmental parameters of the 14 sampling stations in the Bohai Sea. (PDF) [file pone.0061330.s009.pdf]

Table S1. Measurements of *in situ* environmental parameters of the 14 sampling stations in the Bohai Sea.

Environmental factor	Station													
	B1	B3	B5	B7	B8	B10	B11	B12	B14	B15	B16	B19	B20	B22
**Longitude (°E)**	121.50	121.03	120.70	120.01	120.65	118.43	119.48	120.28	118.92	119.17	120.00	119.65	119.90	119.64
**Latitude (°N)**	40.43	40.00	39.67	39.00	38.89	38.45	38.43	38.36	38.23	38.30	38.02	37.92	37.91	37.96
**Water depth (m)**	13.72	29.96	30.26	20.60	37.91	17.90	23.88	28.20	16.91	19.89	18.30	17.10	17.40	17.30
**Surface water (at 1.0 m depth)**														
Temperature (°C)	24.89	24.37	24.72	24.82	22.73	25.43	25.30	23.71	25.83	24.87	23.85	25.21	24.39	24.76
Salinity (PSU[a])	30.04	31.51	31.44	31.31	30.86	31.34	30.51	31.20	31.44	31.54	30.23	30.06	30.15	30.02
EC25[b] (mS/cm)	46.32	48.40	48.27	48.08	47.71	48.05	46.93	48.06	48.15	48.38	46.70	46.31	46.52	46.31
Density (σ_t)	19.63	20.89	20.74	20.61	20.88	20.45	19.87	20.86	20.41	20.77	20.08	19.55	19.86	19.66
Turbidity (FTU[c])	4.38	0.48	0.44	0.32	0.69	5.49	0.71	0.38	12.67	4.97	2.02	3.11	4.78	2.27
Chl-*a* (ppb)	2.47	1.65	1.08	0.94	2.24	2.32	1.96	1.82	2.46	1.91	1.56	2.44	1.55	2.09
**Bottom water**														
Temperature (°C)	24.90	18.94	18.82	23.20	18.79	25.40	20.19	21.95	25.78	24.30	23.80	24.50	24.40	24.01
Salinity (PSU)	30.05	31.61	31.65	31.32	31.12	31.35	31.43	31.21	31.45	31.56	30.24	30.03	30.15	30.15
EC25 (mS/cm)	46.34	49.36	49.45	48.30	48.71	48.08	48.89	48.31	48.16	48.49	46.72	46.35	46.53	46.58
Density (σ_t)	19.64	22.45	22.51	21.09	22.11	20.47	22.00	21.35	20.42	20.96	20.10	19.74	19.86	19.97
Turbidity (FTU)	5.60	6.40	75.52	9.84	5.37	31.42	12.63	3.20	20.33	23.47	7.37	11.17	10.05	6.99
Chl-*a* (ppb)	2.22	1.30	6.12	1.63	0.92	3.91	1.64	1.15	3.10	3.00	1.89	2.23	2.17	1.78
**Sediment**														
Water content (%)	2.50	3.91	23.19	13.93	16.25	32.41	26.65	16.79	5.48	23.44	19.56	1.46	19.32	7.09
Chl-*a* (%)	0.35	0.28	0.77	0.91	2.66	0.12	0.34	0.69	0.29	0.36	0.51	0.69	1.17	0.51
Pha-*a* (%)	1.35	1.22	2.65	2.73	7.24	0.78	1.36	2.23	1.19	1.55	1.99	2.31	3.47	1.99
sulfide[d] (μg/g)	19.50	9.04	12.20	5.40	48.20	7.52	132.0	6.88	29.4	14.8	43.7	103.0	11.3	11.3
OM (organic matter, %)	1.51	2.86	4.38	1.46	3.29	5.79	4.92	2.65	3.74	4.13	2.13	1.11	3.10	3.64
OrgC (organic C, %)	0.63	0.62	0.88	0.89	1.07	2.01	1.41	0.72	1.75	1.82	2.27	1.76	1.15	1.51
OrgN (organic N, %)	0.07	0.05	0.10	0.10	0.07	0.24	0.13	0.02	0.16	0.08	0.22	0.09	0.14	0.07
OrgP (organic P, μmol/g)	1.78	2.68	2.76	2.49	5.19	6.09	5.00	2.06	5.33	5.98	4.10	2.56	4.30	2.78
IP (inorganic P, μmol/g)	9.72	8.17	12.45	12.68	12.63	23.07	18.56	13.47	15.24	18.80	20.10	16.99	21.28	18.08
TP (total P, μmol/g)	11.50	10.85	15.21	15.17	17.82	29.16	23.56	15.53	20.57	24.78	24.20	19.55	25.58	20.86
OrgC/OrgN	9.00	12.40	8.80	8.90	15.29	8.38	10.85	36.00	10.94	22.75	10.32	19.56	8.21	21.57
Petroleum hydrocarbon[d] (μg/g)	14.2	15.1	12.3	20.3	18.2	15.7	16.8	13.3	17.2	17.2	20.9	21.2	15.5	18.6
Hg[d] (ng/g)	50.0	13.0	19.0	6.0	25.0	16.0	22.0	28.0	52.0	57.0	40.0	70.0	43.0	54.0
Cd[d] (μg/g)	0.22	0.19	0.14	0.12	0.12	0.13	0.18	0.09	0.16	0.14	0.13	0.12	0.10	0.12
Pb[d] (μg/g)	25.6	27.0	23.0	21.4	24.0	24.8	22.9	18.0	19.0	24.2	20.8	20.7	18.6	20.2
Cr[d] (μg/g)	49.5	43.4	57.3	45.9	54.4	76.3	76.2	48.8	69.2	75.3	59.2	65.1	53.6	61.7
Zn[d] (μg/g)	70.0	50.6	70.4	48.5	60.7	85.7	87.7	52.6	75.0	85.3	87.7	69.4	55.9	66.6
Cu[d] (μg/g)	19.5	17.2	24.0	13.8	19.4	30.6	29.6	17.6	26.9	30.3	26.7	25.8	18.7	22.5
Co[d] (μg/g)	9.21	5.96	9.19	6.00	7.80	12.90	12.80	7.59	11.50	14.00	8.98	11.30	8.80	9.36
Ni[d] (μg/g)	23.2	19.1	26.6	18.9	22.9	34.3	35.0	20.6	30.5	35.4	22.5	28.2	22.1	26.2
As[d] (μg/g)	5.33	6.13	5.18	5.05	5.98	12.40	8.43	5.58	13.80	14.70	11.50	13.60	13.90	13.30
Fe[d] (mg/g)	21.7	20.0	26.1	20.4	25.8	32.7	36.6	23.8	31.7	35.6	27.0	30.5	26.5	28.6
Mn[d] (μg/g)	399.0	382.0	536.0	594.0	491.0	588.0	676.0	467.0	627.0	684.0	594.0	605.0	621.0	600.0
Sand content (%)	45.08	33.55	17.52	51.65	33.91	1.38	3.31	28.95	3.03	4.09	7.95	15.82	16.98	7.55
Silt content (%)	40.16	50.61	61.27	36.82	46.49	64.23	65.36	59.07	71.67	69.63	77.17	69.31	69.52	75.44
Clay content (%)	14.76	15.84	21.21	11.53	19.60	34.39	31.33	11.98	25.30	26.28	14.88	14.87	13.50	17.01
Median grain size (ø)	5.78	5.38	4.45	6.02	4.76	2.95	3.25	5.51	3.81	3.64	4.94	5.00	5.25	4.80
Mean grain size (ø)	5.00	5.26	5.91	4.60	5.47	6.91	6.73	5.11	6.33	6.40	5.65	5.58	5.38	5.78
Sorting coefficient	1.91	1.90	1.90	1.83	2.02	1.61	1.67	1.73	1.75	1.75	1.67	1.75	1.72	1.74
Kurtosis	2.58	2.32	1.82	3.39	1.91	2.12	2.04	2.94	1.88	1.91	2.50	2.32	2.68	2.18
Skewness	0.97	0.80	0.35	1.28	0.51	-0.15	-0.01	1.04	0.27	0.17	0.80	0.71	0.91	0.64
**Sediment pore-water**														
Salinity (‰)	33.3	34.0	33.8	34.7	34.1	33.9	33.4	33.6	33.3	34.1	33.8	33.6	32.8	33.6
pH	7.71	7.65	7.87	7.95	8.91	7.68	7.72	7.65	7.41	7.65	7.74	7.84	7.66	7.49
Conductivity (mS/cm)	42.6	44.2	43.7	42.0	43.9	43.6	42.6	42.5	43.6	43.9	42.0	41.7	41.3	42.6
Eh (mv)	-38.1	-34.3	-47.2	-51.6	-106.2	-36.2	-38.6	-34.6	-21.6	-34.6	-40.1	-45.8	-34.7	-25.9
DO (μM)	0.21	0.15	0.12	0.17	0.12	0.18	0.10	0.17	0.20	0.09	0.17	0.16	0.18	0.15
NO_3^- (μM)	2.66	1.13	3.92	2.48	4.90	0.07	4.42	1.30	0.29	6.16	6.19	7.34	12.16	20.17
NO_2^- (μM)	1.18	0.86	0.64	1.12	0.93	1.08	0.82	0.94	0.86	0.91	1.08	1.27	1.46	1.55
NO_x^{-e} (μM)	3.84	1.99	4.56	3.6	5.83	1.15	5.24	2.24	1.15	7.07	7.27	8.61	13.62	21.72
NH_4^+ (μM)	257.64	109.08	36.76	57.84	151.36	78.60	59.40	52.82	132.08	124.82	68.48	134.94	88.22	63.24
DIN[f] (μM)	261.48	111.07	41.32	61.44	157.19	79.75	64.64	55.06	133.23	131.89	75.75	143.55	101.84	84.96
NO_2^-/NH_4^+ (%)	0.46	0.79	1.74	1.94	0.61	1.37	1.38	1.78	0.65	0.73	1.58	0.94	1.65	2.45
NO_x^-/NH_4^+ (%)	1.49	1.82	12.40	6.22	3.85	1.46	8.82	4.24	0.87	5.66	10.62	6.38	15.44	34.35
PO_4^{3-} (μM)	2.80	1.92	1.62	4.67	2.31	0.92	1.26	3.31	0.51	1.75	2.86	3.46	1.80	1.58
N/P (DIN/PO_4^{3-})	93.39	57.85	25.51	13.16	68.05	86.68	51.30	16.63	261.24	75.37	26.49	41.49	56.58	53.77
SiO_3^{2-} (μM)	113.43	94.44	87.02	131.65	153.77	71.04	79.75	137.47	101.61	114.19	99.05	189.21	105.46	130.08
SO_4^{2-d} (mg/g)	1.11	1.13	1.35	1.27	1.20	1.92	1.85	1.03	1.24	1.68	0.98	1.02	1.01	1.35

[a] PSU: Practical Salinity Unit;
[b] EC25: Electrical Conductivity calibrated at water temperature of 25 ºC;
[c] FTU: Formazin Turbidity Unit;
[d] These environmental parameters were determined and provided by the Qingdao Branch of the Pony Testing International Group (Qingdao, China);
[e] NO_x^- was calculated as the sum of NO_2^- and NO_3^-;
[f] DIN (the total dissolved inorganic N concentration) was calculated as the sum of NH_4^+, NO_2^- and NO_3^-.
