# Supplementary material for: Molecular Detection of Candidatus Scalindua pacifica and Environmental Responses of Sediment Anammox Bacterial Community in the Bohai Sea, China
Source: PLoS One. 2013 Apr 8;8(4):e61330. doi: 10.1371/journal.pone.0061330 (PMC3620062; doi:10.1371/journal.pone.0061330)
Supplement: Table S2 — Correlation analyses of biodiversity and abundance of key microbial groups with Bohai Sea environmental factors. (PDF) [file pone.0061330.s010.pdf]

Table S2. Correlation analyses of biodiversity and abundance of key microbial groups with Bohai Sea environmental factors.

Environmental factor	Pearson correlation coefficient (p value)*												
	Gene biodiversity										Gene abundance		
	Scalindua 16S rRNA gene biodiversity					Anammox *hzo* gene biodiversity					Bacterial 16S rRNA	*Scalindua* 16S rRNA	Anammox *hzo*
	H	J	$1/D$	S_{Chao1}	S_{ACE}	H	J	$1/D$	S_{Chao1}	S_{ACE}			
**Water depth**	-0.504 (0.066)	-0.274 (0.344)	-0.397 (0.160)	-0.320 (0.264)	-0.229 (0.431)	0.331 (0.248)	0.338 (0.237)	0.283 (0.327)	**0.652 (0.012)**	0.265 (0.360)	-0.229 (0.431)	-0.383 (0.177)	-0.223 (0.444)
**Surface water**													
Temperature	0.027 (0.928)	-0.290 (0.315)	-0.079 (0.788)	0.255 (0.379)	0.239 (0.411)	-0.238 (0.413)	-0.226 (0.437)	-0.107 (0.715)	**-0.630 (0.016)**	-0.279 (0.335)	0.027 (0.926)	**0.761 (0.002)**	**0.649 (0.012)**
Salinity	**-0.638 (0.014)**	**-0.597 (0.024)**	**-0.588 (0.027)**	-0.291 (0.312)	-0.119 (0.686)	-0.065 (0.825)	0.023 (0.939)	-0.062 (0.833)	-0.081 (0.783)	-0.229 (0.431)	**-0.579 (-0.579)**	0.006 (0.985)	0.108 (0.714)
EC25	**-0.646 (0.013)**	**-0.571 (0.033)**	**-0.584 (0.028)**	-0.319 (0.266)	-0.144 (0.623)	-0.041 (0.890)	0.046 (0.876)	-0.051 (0.862)	-0.012 (0.969)	-0.199 (0.495)	**-0.582 (0.029)**	-0.074 (0.802)	0.039 (0.894)
Density	**-0.603 (0.022)**	-0.421 (0.133)	-0.509 (0.063)	-0.385 (0.174)	-0.219 (0.452)	0.048 (0.870)	0.125 (0.670)	-0.008 (0.978)	0.210 (0.471)	-0.087 (0.767)	**-0.550 (0.041)**	-0.344 (0.228)	-0.198 (0.497)
Turbidity	-0.044 (0.881)	-0.223 (0.444)	-0.005 (0.987)	0.073 (0.805)	0.107 (0.717)	**-0.550 (0.041)**	**-0.702 (0.005)**	-0.508 (0.064)	-0.198 (0.498)	-0.042 (0.886)	0.180 (0.538)	**0.633 (0.015)**	**0.548 (0.043)**
Chl-*a*	-0.198 (0.498)	-0.255 (0.379)	-0.249 (0.391)	0.155 (0.597)	0.153 (0.602)	-0.301 (0.296)	-0.446 (0.110)	-0.244 (0.400)	0.173 (0.555)	0.100 (0.734)	**0.605 (0.022)**	0.512 (0.061)	0.280 (0.332)
**Bottom water**													
Temperature	0.423 (0.131)	0.219 (0.451)	0.334 (0.243)	0.270 (0.350)	0.244 (0.400)	**-0.596 (0.024)**	**-0.590 (0.027)**	**-0.584 (0.028)**	**-0.588 (0.027)**	-0.391 (0.167)	0.282 (0.328)	0.362 (0.204)	0.160 (0.586)
Salinity	**-0.664 (0.010)**	**-0.667 (0.009)**	**-0.636 (0.014)**	-0.244 (0.401)	-0.087 (0.768)	0.071 (0.808)	0.149 (0.612)	0.101 (0.730)	0.062 (0.832)	-0.071 (0.810)	-0.503 (0.067)	0.069 (0.814)	0.141 (0.630)
EC25	**-0.679 (0.008)**	**-0.619 (0.018)**	**-0.625 (0.017)**	-0.281 (0.330)	-0.143 (0.625)	0.253 (0.384)	0.308 (0.283)	0.273 (0.346)	0.248 (0.393)	0.074 (0.801)	-0.493 (0.073)	-0.055 (0.851)	0.072 (0.808)
Density	**-0.598 (0.024)**	-0.461 (0.097)	-0.527 (0.053)	-0.300 (0.297)	-0.209 (0.472)	0.442 (0.113)	0.479 (0.083)	0.448 (0.108)	0.423 (0.132)	0.227 (0.435)	-0.434 (0.121)	-0.220 (0.450)	-0.050 (0.865)
**Sediment**													
Chl-*a*	0.086 (0.771)	0.129 (0.660)	0.153 (0.601)	-0.134 (0.647)	-0.043 (0.883)	-0.059 (0.840)	-0.039 (0.894)	-0.170 (0.560)	**0.721 (0.004)**	0.157 (0.592)	0.288 (0.319)	-0.452 (0.105)	-0.411 (0.144)
Pha-*a*	0.095 (0.748)	0.142 (0.629)	0.160 (0.586)	-0.118 (0.687)	-0.036 (0.903)	-0.064 (0.828)	-0.044 (0.882)	-0.173 (0.554)	**0.714 (0.004)**	0.157 (0.592)	0.269 (0.353)	-0.467 (0.092)	-0.419 (0.136)
OM	-0.280 (0.332)	**-0.600 (0.023)**	-0.272 (0.346)	0.410 (0.145)	**0.536 (0.048)**	-0.116 (0.693)	-0.218 (0.455)	-0.064 (0.828)	0.060 (0.838)	0.004 (0.989)	0.003 (0.992)	0.392 (0.166)	0.434 (0.121)
OrgC	0.155 (0.597)	0.181 (0.536)	0.087 (0.766)	0.170 (0.562)	0.191 (0.512)	**-0.575 (0.031)**	**-0.586 (0.028)**	**-0.574 (0.032)**	-0.201 (0.490)	0.002 (0.994)	-0.076 (0.795)	0.394 (0.163)	0.202 (0.488)
IP	0.511 (0.062)	0.301 (0.295)	0.462 (0.096)	0.397 (0.160)	0.428 (0.127)	-0.513 (0.061)	**-0.532 (0.050)**	**-0.542 (0.045)**	-0.149 (0.611)	-0.062 (0.833)	0.221 (0.447)	0.366 (0.199)	0.202 (0.490)
TP	0.379 (0.181)	0.165 (0.574)	0.343 (0.229)	0.345 (0.227)	0.417 (0.138)	-0.526 (0.053)	**-0.561 (0.037)**	**-0.542 (0.045)**	-0.149 (0.611)	-0.062 (0.833)	0.196 (0.502)	0.388 (0.170)	0.225 (0.440)
Hg	0.354 (0.214)	0.301 (0.295)	0.270 (0.351)	0.133 (0.651)	-0.042 (0.888)	-0.503 (0.067)	**-0.541 (0.046)**	-0.426 (0.129)	-0.237 (0.414)	-0.124 (0.673)	0.409 (0.147)	0.266 (0.359)	0.100 (0.734)
Cd	-0.462 (0.096)	-0.468 (0.092)	-0.472 (0.088)	-0.124 (0.672)	-0.145 (0.621)	**0.579 (0.030)**	0.461 (0.097)	**0.661 (0.010)**	0.143 (0.626)	0.419 (0.136)	-0.069 (0.814)	0.158 (0.588)	0.183 (0.531)
Pb	-0.529 (0.052)	**-0.540 (0.046)**	**-0.554 (0.040)**	-0.061 (0.835)	0.071 (0.809)	**0.555 (0.039)**	**0.534 (0.049)**	**0.563 (0.036)**	0.287 (0.320)	0.213 (0.464)	-0.162 (0.580)	-0.167 (0.569)	-0.095 (0.747)
Cr	-0.038 (0.898)	-0.282 (0.329)	-0.165 (0.573)	0.292 (0.310)	0.342 (0.231)	-0.490 (0.075)	-0.471 (0.089)	-0.401 (0.155)	-0.196 (0.503)	-0.113 (0.702)	0.159 (0.587)	**0.617 (0.019)**	0.370 (0.193)
Cu	-0.156 (0.593)	-0.247 (0.395)	-0.245 (0.399)	0.153 (0.601)	0.189 (0.518)	-0.331 (0.247)	-0.342 (0.231)	-0.249 (0.390)	-0.174 (0.551)	0.034 (0.907)	-0.023 (0.937)	**0.551 (0.041)**	0.345 (0.227)
Co	-0.017 (0.954)	-0.280 (0.333)	-0.151 (0.606)	0.225 (0.440)	0.258 (0.373)	-0.440 (0.116)	-0.407 (0.148)	-0.337 (0.239)	-0.271 (0.348)	-0.163 (0.577)	0.238 (0.413)	**0.594 (0.025)**	0.320 (0.265)
Ni	-0.137 (0.640)	-0.440 (0.116)	-0.278 (0.337)	0.279 (0.334)	0.337 (0.239)	-0.395 (0.162)	-0.360 (0.207)	-0.282 (0.329)	-0.216 (0.458)	-0.164 (0.575)	0.173 (0.555)	**0.611 (0.020)**	0.377 (0.184)
As	0.440 (0.116)	0.264 (0.362)	0.404 (0.152)	0.342 (0.231)	0.290 (0.315)	**-0.684 (0.007)**	**-0.785 (0.001)**	**-0.646 (0.013)**	-0.318 (0.268)	-0.126 (0.668)	0.227 (0.434)	0.521 (0.056)	0.416 (0.139)
Fe	0.040 (0.892)	-0.189 (0.517)	-0.077 (0.793)	0.252 (0.385)	0.267 (0.356)	-0.494 (0.073)	-0.479 (0.083)	-0.400 (0.156)	-0.144 (0.624)	-0.071 (0.810)	0.213 (0.465)	**0.589 (0.027)**	0.341 (0.233)
Mn	0.448 (0.108)	0.172 (0.556)	0.323 (0.261)	0.278 (0.336)	0.268 (0.355)	**-0.633 (0.015)**	-0.526 (0.053)	**-0.586 (0.028)**	-0.334 (0.243)	-0.245 (0.399)	0.076 (0.797)	0.386 (0.173)	0.193 (0.508)
Sand content	-0.058 (0.845)	0.056 (0.850)	-0.040 (0.891)	-0.314 (0.274)	-0.285 (0.322)	0.442 (0.113)	**0.556 (0.039)**	0.368 (0.196)	0.189 (0.519)	-0.065 (0.826)	-0.008 (0.979)	**-0.602 (0.023)**	-0.519 (0.057)
Silt content	0.289 (0.316)	0.292 (0.312)	0.301 (0.296)	0.267 (0.357)	0.145 (0.620)	-0.470 (0.090)	**-0.594 (0.025)**	-0.415 (0.140)	-0.260 (0.370)	0.064 (0.828)	-0.014 (0.961)	0.458 (0.100)	0.424 (0.130)
Clay content	-0.383 (0.177)	**-0.647 (0.012)**	-0.443 (0.113)	0.243 (0.402)	0.394 (0.163)	-0.173 (0.553)	-0.215 (0.459)	-0.103 (0.727)	0.031 (0.915)	0.034 (0.907)	0.043 (0.883)	**0.562 (0.037)**	0.432 (0.123)
Median grain size	0.280 (0.332)	0.499 (0.069)	0.331 (0.247)	-0.247 (0.395)	-0.360 (0.207)	0.312 (0.278)	0.362 (0.204)	0.239 (0.410)	0.011 (0.970)	-0.031 (0.917)	-0.028 (0.923)	**-0.596 (0.025)**	-0.459 (0.099)
Mean grain size	-0.232 (0.424)	-0.442 (0.114)	-0.279 (0.334)	0.285 (0.323)	0.364 (0.200)	-0.293 (0.309)	-0.370 (0.193)	-0.210 (0.470)	-0.059 (0.842)	0.059 (0.840)	0.034 (0.907)	**0.634 (0.015)**	0.511 (0.062)
Sorting coefficient	-0.366 (0.198)	-0.289 (0.316)	-0.310 (0.280)	-0.259 (0.370)	-0.221 (0.448)	0.372 (0.191)	0.375 (0.186)	0.343 (0.230)	**0.556 (0.039)**	0.164 (0.575)	-0.025 (0.934)	-0.496 (0.071)	-0.311 (0.280)
Kurtosis	0.441 (0.115)	**0.544 (0.045)**	0.428 (0.127)	-0.161 (0.583)	-0.200 (0.492)	0.135 (0.646)	0.284 (0.325)	0.049 (0.867)	-0.325 (0.258)	-0.266 (0.358)	-0.031 (0.916)	-0.403 (0.153)	-0.433 (0.122)
Skewness	0.371 (0.191)	**0.591 (0.026)**	0.414 (0.141)	-0.244 (0.400	-0.364 (0.201)	0.212 (0.466)	0.280 (0.333)	0.134 (0.649)	-0.083 (0.779)	-0.079 (0.787)	-0.044 (0.882)	**-0.567 (0.034)**	-0.467 (0.092)
**Sediment pore-water**													
Salinity	-0.335 (0.241)	-0.213 (0.465)	-0.446 (0.110)	-0.136 (0.643)	-0.005 (0.987)	-0.020 (0.946)	0.236 (0.417)	-0.071 (0.809)	-0.025 (0.932)	-0.323 (0.260)	**-0.540 (0.046)**	-0.477 (0.085)	-0.469 (0.091)
pH	-0.181 (0.535)	-0.066 (0.822)	-0.162 (0.579)	-0.240 (0.408)	-0.086 (0.771)	0.107 (0.716)	0.192 (0.511)	0.001 (0.997)	**0.748 (0.002)**	0.191 (0.514)	0.143 (0.625)	-0.465 (0.094)	-0.491 (0.075)
Conductivity	**-0.829 (0.000)**	**-0.828 (0.000)**	**-0.791 (0.001)**	-0.047 (0.874)	0.089 (0.763)	0.089 (0.762)	0.014 (0.963)	0.128 (0.664)	0.304 (0.290)	0.088 (0.765)	-0.321 (0.263)	0.029 (0.922)	0.167 (0.568)
Eh	0.184 (0.528)	0.067 (0.820)	0.167 (0.569)	0.240 (0.409)	0.087 (0.768)	-0.101 (0.732)	-0.186 (0.524)	0.005 (0.988)	**-0.747 (0.002)**	-0.191 (0.514)	-0.142 (0.627)	0.462 (0.096)	0.490 (0.075)
NO_3^-	**0.723 (0.003)**	0.445 (0.111)	**0.643 (0.013)**	**0.685 (0.007)**	0.494 (0.073)	-0.274 (0.344)	-0.346 (0.226)	-0.244 (0.402)	-0.087 (0.768)	-0.125 (0.670)	0.396 (0.161)	-0.102 (0.728)	-0.013 (0.965)
NO_2^-	**0.813 (0.000)**	**0.575 (0.031)**	**0.751 (0.002)**	**0.567 (0.034)**	0.452 (0.105)	-0.299 (0.299)	-0.368 (0.196)	-0.329 (0.251)	-0.236 (0.417)	-0.228 (0.433)	**0.565 (0.035)**	-0.003 (0.993)	-0.022 (0.940)
NO_x^-	**0.736 (0.003)**	0.456 (0.101)	**0.656 (0.011)**	**0.688 (0.007)**	0.498 (0.070)	-0.278 (0.336)	-0.351 (0.219)	-0.250 (0.388)	-0.095 (0.748)	-0.131 (0.654)	0.408 (0.147)	-0.099 (0.736)	-0.013 (0.964)
N/P	-0.326 (0.255)	-0.449 (0.107)	-0.264 (0.362)	0.009 (0.974)	0.050 (0.866)	-0.382 (0.178)	**-0.581 (0.029)**	-0.327 (0.254)	0.074 (0.801)	0.151 (0.607)	0.136 (0.643)	**0.609 (0.021)**	**0.577 (0.031)**
NO_x^-/NH_4^+	**0.603 (0.023)**	0.320 (0.265)	0.526 (0.053)	**0.748 (0.002)**	**0.557 (0.038)**	-0.188 (0.519)	-0.245 (0.398)	-0.145 (0.620)	-0.183 (0.531)	-0.164 (0.576)	0.191 (0.513)	-0.103 (0.726)	0.048 (0.870)
PO_4^{3-}	0.268 (0.354)	0.523 (0.055)	0.186 (0.523)	-0.360 (0.207)	-0.400 (0.157)	0.113 (0.701)	0.388 (0.171)	0.036 (0.903)	-0.160 (0.586)	-0.279 (0.334)	-0.110 (0.709)	**-0.589 (0.027)**	**-0.692 (0.006)**
SO_4^{2-}	-0.221 (0.447)	**-0.614 (0.020)**	-0.370 (0.193)	0.415 (0.141)	**0.549 (0.042)**	-0.125 (0.669)	-0.052 (0.861)	-0.049 (0.867)	-0.155 (0.597)	-0.231 (0.427)	0.043 (0.885)	0.345 (0.226)	0.215 (0.461)

* The colored boxes show the Pearson correlation coefficients and p values that are significant ($\leq$ 0.05) for positive (yellow-colored) or negative correlations (blue-colored). The environmental factors that did not have any significant correlation with microbial biodiversity or abundance were not shown in this table.
