# Supplementary material for: Molecular Detection of Candidatus Scalindua pacifica and Environmental Responses of Sediment Anammox Bacterial Community in the Bohai Sea, China
Source: PLoS One. 2013 Apr 8;8(4):e61330. doi: 10.1371/journal.pone.0061330 (PMC3620062; doi:10.1371/journal.pone.0061330)
Supplement: Table S3 — Efficiency and sensitivity of individual qPCR standard curve determined via linearized plasmid DNA. (PDF) [file pone.0061330.s011.pdf]

Table S3. Efficiency and sensitivity of individual qPCR standard curve determined via linearized plasmid DNA.

Target gene	Primer set	Efficiency			Sensitivity (copy number/µl plasmid DNA)	
		R^2	Slope	Linearity range	Expected value	Obtained value
Bacterial 16S RNA	341F, 518R	0.991	-3.365	10^3-10^9	1.870 x 10^6	1.837 x 10^6
Scalindua 16S rRNA	Brod541F, Brod1260R	0.998	-3.367	10^1-10^6	2.935 x 10^6	2.877 x 10^6
Anammox bacterial *hzo*	HzoF1, HzoR1	0.996	-3.266	10^1-10^6	3.289 x 10^6	3.367 x 10^6
